# Index matching improves the imaging quality of 3D well-of-the-well dishes for embryo culture

**DOI:** 10.1117/1.BIOS.3.1.012103

**Published:** 2026-01-07

**Authors:** Yunqin Zhao, Mark Mc Veigh, Leon M. Bellan, Audrey K. Bowden

**Affiliations:** a Vanderbilt University, Vanderbilt Biophotonics Center, Department of Biomedical Engineering, Nashville, Tennessee, United States; b Vanderbilt University, Department of Biomedical Engineering, Nashville, Tennessee, United States; c Vanderbilt University, Interdisciplinary Materials Science Program, Nashville, Tennessee, United States; d Vanderbilt University, Department of Mechanical Engineering, Nashville, Tennessee, United States; e Vanderbilt University, Department of Electrical and Computer Engineering, Nashville, Tennessee, United States

**Keywords:** *in vitro* fertilization, embryo culture, embryo selection, embryo grading, embryo imaging, 3D culture

## Abstract

**Significance:**

The success rate of *in vitro* fertilization (IVF) is low (<33%), driving the need for techniques to improve embryo quality. One promising solution is the well-of-the-well (WOW) culture system. WOWs with 3D microwells offer superior culture quality over 2D microwells; however, the refractive index (RI) mismatch between the 3D microwells and culture media causes aberrations that limit imaging quality and hinder clinical translation.

**Aim:**

To enable clinical deployment of WOW dishes, we demonstrate the fabrication of index-matching 3D WOW dishes that improve embryo imaging quality.

**Approach:**

We applied 3D printing and molding to fabricate agarose-based WOW dishes. The fabricated product was inspected using optical coherence tomography, and its reproducibility was validated. We applied a slanted-edge method to characterize optical resolution through substrates with different RIs and assessed geometric distortion of WOW dishes with different RIs using certified microspheres. To evaluate additional RI-dependent optical aberrations, we performed Shack–Hartmann wavefront sensing and Zernike analysis on WOW dishes of different RIs. Finally, we assessed the stability of agarose microwells after a 6-day incubation and demonstrated *in situ* imaging using mouse embryos.

**Results:**

We confirmed that the proposed fabrication method has acceptable reproducibility. We found that low-RI material mitigates spherical aberration, improves optical resolution compared with high-RI materials, reduces geometric distortion, and eliminates visibility of ridge structures. Consistent with these observations, wavefront analysis showed that high-RI materials generated substantially higher-order aberrations, whereas low-RI materials produced minimal higher-order error. We validated that the agarose microwells retained their shapes after a 6-day incubation. We confirmed normal development of mouse embryos over 4 days in agarose-based dishes, indicating agarose-based WOW dishes are feasible for embryo culture.

**Conclusions:**

The proposed fabrication strategy eliminates a barrier to the integration of 3D WOW dishes for both culture and selection and may enable their clinical use to enhance IVF success rates.

Statement of Discovery This work introduces a method to fabricate index-matching 3D well-of-the-well (WOW) dishes to improve the imaging quality of embryos viewed directly in microwells. The proposed fabrication strategy eliminates a barrier to the integration of 3D WOW dishes for both culture and selection, which may enable their clinical use to enhance IVF success rates.

## 1 Introduction

Infertility is a prevalent condition that globally affects 15% of couples of reproductive age (ages 15 to 44).^1^ Despite the availability of assisted reproductive technology (ART) to treat infertility, the average success rate of ART in the US is a paltry 33%.^2^ The culture and selection of embryos during *in vitro* fertilization (IVF), the most common form of ART, are crucial steps that can affect embryo quality and determine the success of the procedure.^3^ Unfortunately, current processes for embryo culture and selection in IVF are inadequate to enable strong success rates.^4^^–^^6^

**Fig. 1:**
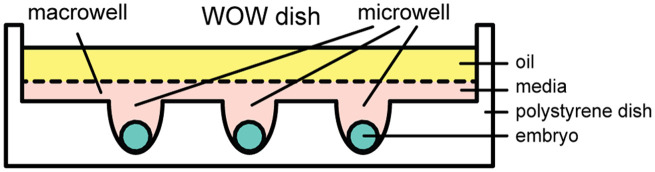
Embryos cultured in a WOW dish are isolated in confined spaces termed microwells but share a small volume of culture media and overlay oil that fills the macrowell. The example WOW dish shown here includes three 3D microwells of semi-ellipsoidal shape. Note: not drawn to scale. Adapted from Ref. 18 under a Creative Commons Attribution-NonCommercial-NoDerivatives 4.0 International License. Used with permission provided by Springer Nature.

Culture conditions may be one determinant of embryo quality. Many factors, such as oxygen concentration,^7^^,^^8^ culture media pH,^9^ and temperature,^10^^–^^12^ impact the embryo culture quality. The choice of culture dish is another factor that can affect the culture quality. Most IVF procedures involve the culture of embryos in IVF dishes, which resemble standard petri dishes. However, previous studies have shown that embryos grown in well-of-the-well (WOW) dishes,^6^^,^^13^ an *in-vitro* embryo culture platform, have better quality than those grown in IVF dishes, suggesting that embryo culture in WOW dishes may improve IVF outcomes.^15^^–^^17^ A WOW dish (Fig. 1) comprises two types of wells to confine media and embryos, respectively: a single, larger well, which we term a macrowell, and several smaller wells, which we term microwells. The microwells can take on different shapes, including semi-elliptical and truncated conical, and may have flat or nonflat bottoms. Among WOW dish designs, it has been shown that 3D WOW dishes (i.e., those having microwells with nonflat bottoms) yield better quality embryos than those with 2D flat-bottomed microwells (e.g., truncated cones).^18^

**Fig. 2:**
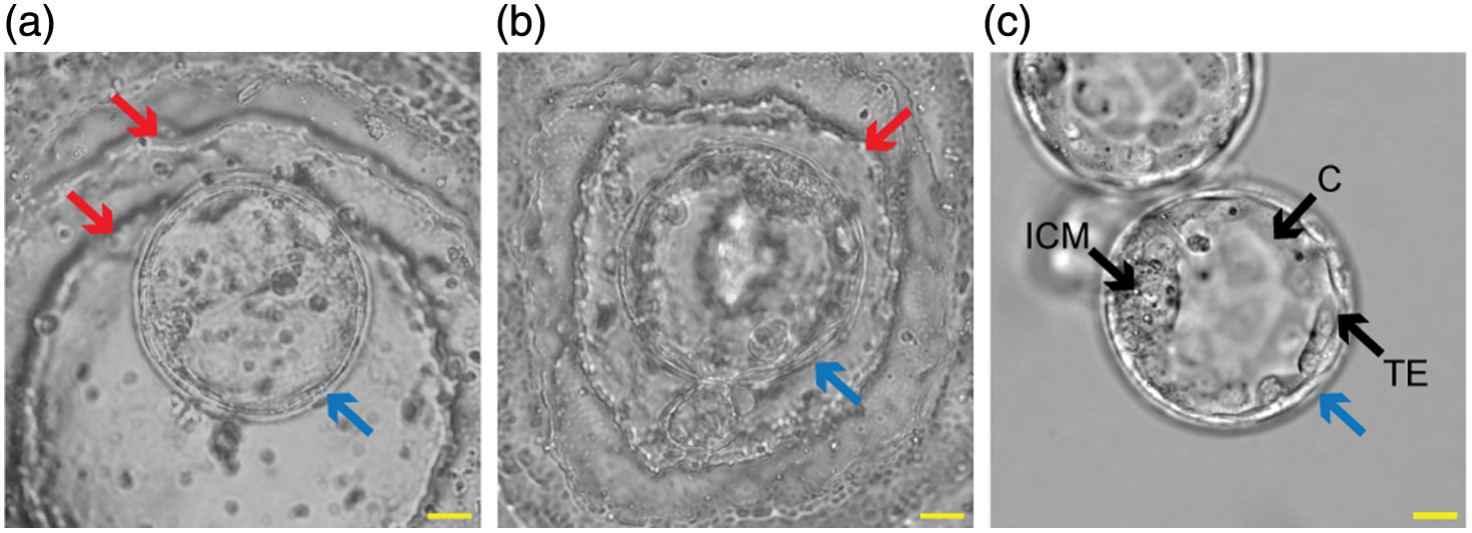
Brightfield images of mouse embryo blastocysts that are imaged in a (a) hemispherical microwell and (b) pyramidal microwell well-of-the-well (WOW) dish made of polydimethylsiloxane (PDMS)^18^ and an (c) IVF dish made of polystyrene. Cellular structures labeled include inner cell mass (ICM), trophectoderm (TE), and cavity (C). Ridges on the PDMS WOW dishes induced during fabrication are visible in the images and indicated by red arrows. Embryo boundaries are indicated by blue arrows. (Scale bar: 20  μm).

Despite their potential for improving embryo quality, a major barrier to the clinical adoption of 3D WOW dishes is their limited utility for embryo grading—a critical step in the selection process. During embryo grading, embryologists image the cultured embryos using an inverted brightfield (BF) microscope (i.e., *in situ* imaging) and employ their clinical judgement to assess the embryo quality according to a grading system.^19^ This morphological assessment requires clear and undistorted visualization of cellular structures as low-quality embryo images have been shown to lead to disagreements in embryo grading between embryologists.^20^ Unfortunately, current 3D WOW dishes exhibit several sample-induced optical aberrations when viewed with BF, leading to low-quality images of the embryo that make them challenging to grade accurately. Example images of a mouse blastocyst in a hemispherical and a pyramidal microwell made of polydimethylsiloxane (PDMS) are shown in Figs. 2(a) and 2(b), respectively. In the images, the subcellular structures of the blastocyst, including the inner cell mass (ICM) and trophectoderm (TE), with an appearance that is key to embryo grading, are hard to visualize due to optical aberrations and the visibility of ridges introduced during fabrication of the WOW dish. By contrast, embryos viewed in flat-bottom dishes, including IVF dishes and flat WOW dishes, suffer fewer aberrations and offer clear visualization of the ICM and TE for embryo grading [Fig. 2(c)].

Several aberrations arise from refractive index (RI) mismatch between the dish (RI∼1.58 for polystyrene^6^^,^^13^^,^^15^), the microwell structures (e.g., RI∼1.41 for PDMS^18^), and the culture medium (RI 1.334 to 1.345 depending on formulation). The embryo culture medium (MR-101-D, Sigma Aldrich, St. Louis, Missouri, United States) used in this study has an RI of 1.335, as measured with a refractometer (75997-572, VWR).

**Fig. 3:**
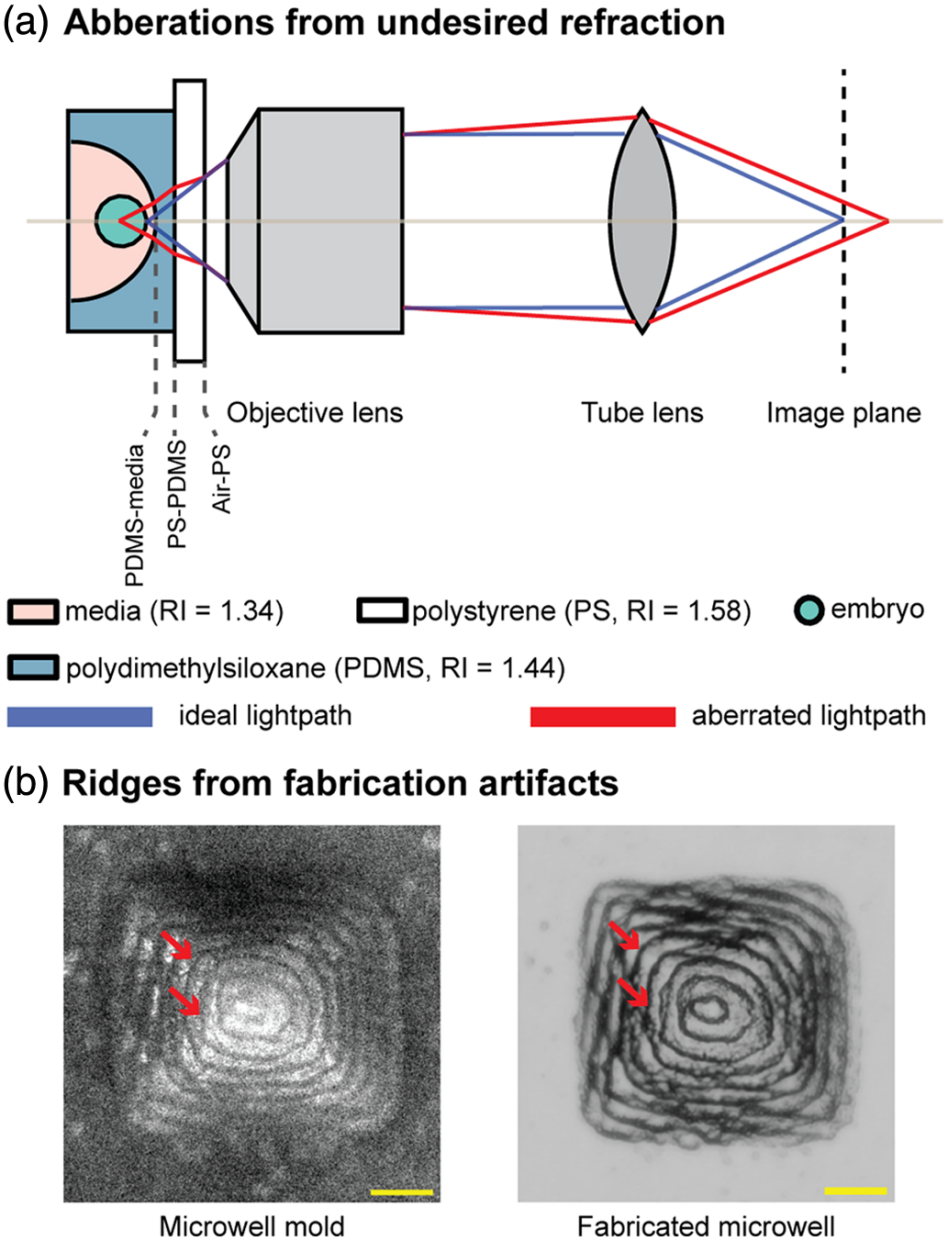
Sources of aberrations of 3D microwell imaging: (a) Spherical and defocus aberrations due to the media and dish refractive index (RI) mismatch as well as the microwell shape lead to inaccurate focusing of the light path at the image plane; (b) ridges due to the fabrication artifact in a microwell mold and its fabricated product hinder visibility of the embryo and subcellular structures (scale bar: 100  μm). Panel (b) adapted from Ref. 18 under a Creative Commons Attribution-NonCommercial-NoDerivatives 4.0 International License. Used with permission provided by Springer Nature.

First, spherical aberration,^21^^,^^22^ observed at the boundaries between air and the dish base (Air-PS) and between the dish base and the microwell structures (PS-PDMS), impairs the ability of light to focus according to the optical design of the objective, thereby worsening resolution and image clarity. Second, geometric distortion arises from microwell-shape–dependent refraction, where microwell geometries such as semi-elliptical or pyramidal wells bend light in ways that distort embryo features. Third, undesired refraction at the microwell–medium boundary (PDMS-media) introduces additional aberrations, including spherical aberration, field curvature, defocus, coma, astigmatism, trefoil, tetrafoil, and quatrefoil, all of which further degrade imaging resolution and clarity. Figure 3(a) illustrates the effects of on-axis aberrations, including spherical aberration and defocus, which induce focal shifts that reduce image sharpness. Off-axis aberrations, including field curvature, coma, astigmatism, trefoil, tetrafoil, and quatrefoil, which typically produce edge blurring and field-dependent geometric distortion, also impact imaging performance; however, they are not depicted because their field-dependent nature requires more comprehensive modeling than is appropriate for a conceptual illustration.

Finally, fabrication artifacts such as ridges caused by limited printing resolution can also degrade image quality. As shown in Figs. 2(a), 2(b), and 3(b), ridges from the 3D-printed mold^18^ are replicated in the fabricated microwell and may overlap with the embryo, interrupting visibility and hindering grading.

The problems caused by refractive index mismatch during cell imaging are not isolated to WOW dishes. In his work with HeLa and zebrafish embryos, Boothe et al.^22^ showed that RI matching is a viable strategy to mitigate distortion and improve optical resolution in cell culture; however, the iodixanol that was added to the culture media to change the RI is cytotoxic and, although safe for zebrafish, is not ideal for human embryo culture in IVF due to its excess oxidative stress^23^ that may harm embryos. In addition, although fabricating cell culture dishes from optical epoxies with an RI that is close to water, such as BIO-133/BIO-134 (MY Polymers, Ness Ziona, Israel), has been shown to improve imaging during cell culture,^24^ optical epoxies are also cytotoxic to embryos and are, therefore, not suitable for IVF. In our preliminary study (data not shown), mouse embryos that were cultured on a BIO-133 film stopped developing at the one-cell stage. Importantly, mouse embryos are used as part of quality control assays for human IVF.^25^^,^^26^

The purpose of this study is to introduce a new method to fabricate WOW dishes that are free from the optical aberrations that currently degrade imaging quality. As an alternative to the above materials, we propose hydrogels as a suitable material for the fabrication of RI-matched culture dishes to support mouse and human embryo culture. Hydrogels have been widely employed as a biocompatible extracellular matrix (ECM) for 3D cell culture to recapitulate native cellular milieus.^27^ Agarose gel, a common hydrogel, has been used previously to fabricate microwell plates to culture cells;^28^^–^^30^ however, its application for use in WOW dishes to produce microwells of various shapes has not been demonstrated. In addition, the optical performance of using agarose microwells has not been studied before. In this study, we demonstrate the first use of agarose [RI = 1.335 (1.5% (w/v%)^31^)] to fabricate 3D WOW dishes, enabling a near exact RI match with the desired culture media for embryos during IVF. Moreover, we confirm the successful growth and healthy development of mouse embryos over 4 days in agarose-based dishes, indicating that the dish is feasible for embryo culture. In addition, we explored the degree to which the application of a low-RI material (i.e., agarose) mitigates spherical aberration and improves the optical resolution of imaged objects compared with high-RI materials (i.e., PDMS), reduces geometrical distortion, and eliminates visibility of fabricated ridge structures. To evaluate additional RI-dependent optical aberrations, we performed Shack–Hartmann wavefront sensing and Zernike analysis on WOW dishes of different RIs. We also confirmed the reproducibility of the proposed fabrication method and verified the integrity of the agarose microwell after 6-day incubation.

The proposed strategy of utilizing agarose to make WOW dishes for embryo culture is the first to demonstrate BF imaging of embryos in 3D WOW dishes with uncorrupted imaging quality. It thus offers a route to improve the grading and selection of embryos grown in WOW dishes when visualized with standard IVF tools. The ability to image 3D WOW dishes with standard IVF tools could pave the way for broader adoption of WOW dishes by the IVF community, which could lead to improved embryo growth and outcomes for IVF in general. Overall, this strategy to eliminate optical aberrations will also make it possible to explore the utility of WOW dishes with more complex microwell shapes, which may support even better embryo growth potential.^18^^,^^32^

## 2 Methods

### 2.1 Agarose WOW Dish Design and Fabrication

**Fig. 4:**
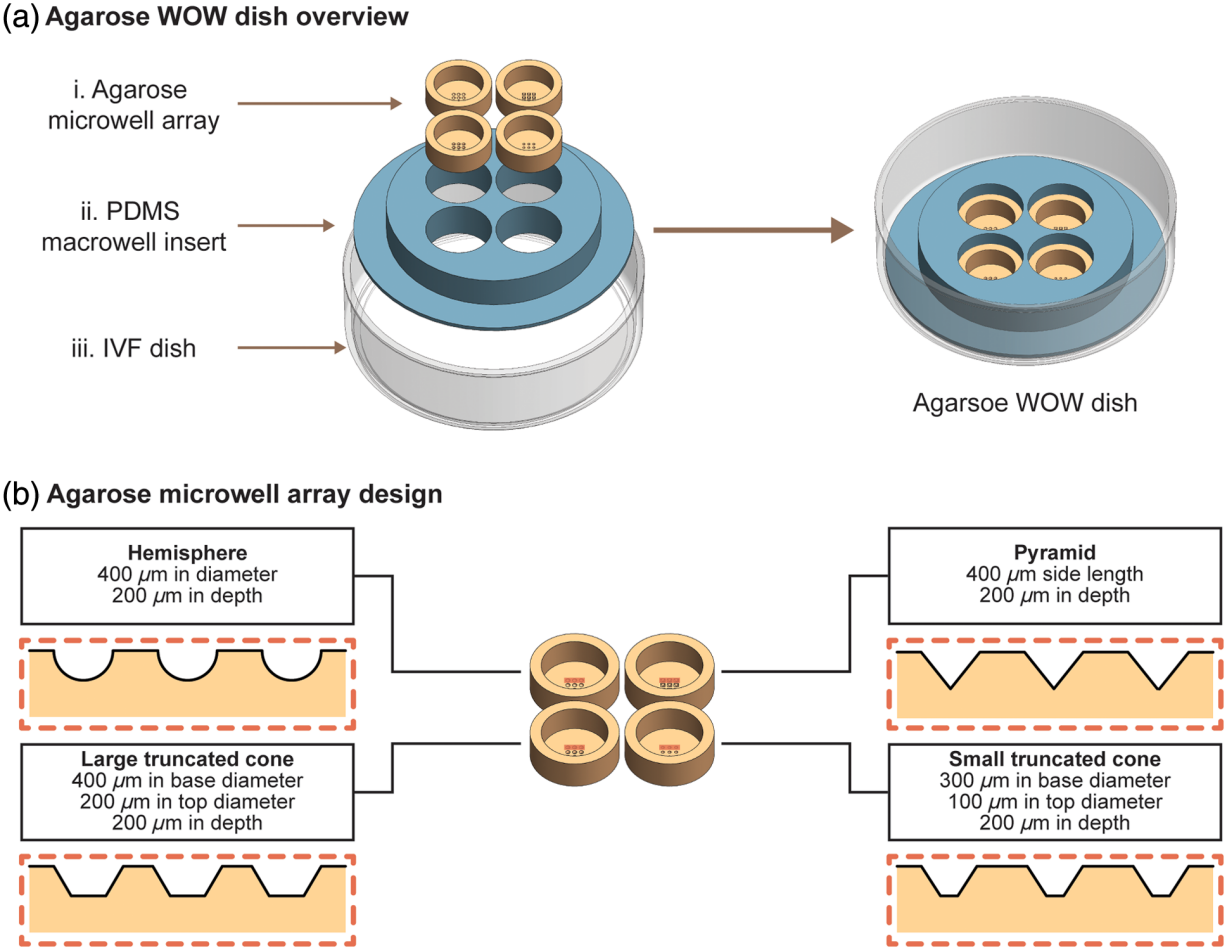
(a) Overview of an agarose well-of-the-well (WOW) dish comprising four agarose microwell arrays (i), a PDMS macrowell insert (ii), and an IVF dish (iii). (b) Agarose microwell array designs with cross-sectional views. Not drawn to scale.

Our fabrication strategy involves the use of 3D printing and molding to convert an IVF dish into a 3D WOW. The WOW dishes we fabricate comprise three components (Fig. 4): a polystyrene IVF dish, a PDMS macrowell insert, and an agarose microwell array. In our current design, the PDMS macrowell insert holds four different agarose microwell arrays. The combination of the PDMS macrowell insert and the agarose microwell array forms the WOW structure. The IVF dish provides a stable bottom support for the WOW dish, and the vented lid that comes with the dish facilitates air exchange during culturing. The PDMS macrowell insert serves as a fence around the agarose microwell array, both to immobilize the array and to hold the culture media. The fence feature is necessary because agarose is water-permeable, so the agarose microwell array cannot, on its own, hold the culture media necessary to sustain embryo growth. The PDMS macrowell insert also controls the volume of culture media accessible to the embryo, which has been shown to affect blastocyst formation.^33^ Each agarose microwell array, with RI that is matched to the media, contains impressions forming the microwells for embryo culture; the microwells may be the same or differ in shape and size depending on the research protocol. In our current experiment, each microwell array has nine impressions identical in shape and dimensions, each of which can hold a single embryo; however, each of the four microwell arrays features a different microwell shape [Fig. 4(b)]. Note that the microwell shape and size are critical factors that affect embryo growth outcomes.^18^

#### 2.1.1 PDMS macrowell insert fabrication

We designed a negative macrowell mold in SolidWorks (Dassault Systèmes, Vélizy-Villacoublay, France) to template a PDMS macrowell insert containing four cylindrical wells (height: 4 mm, diameter: 8 mm). A complete drawing of the macrowell mold is included in Fig. S1 in the Supplementary Material. The mold was then printed by a resin 3D printer (Phrozen Sonic Mini 8K, Phrozen Tech) and coated with 2.5 g parylene C (DPX-C, Specialty Coating Systems) to prevent unreacted cytotoxic resin on the mold from entering PDMS and to prevent adhesion of the mold to PDMS. The detailed coating protocol is described elsewhere.^34^

PDMS (SYLGARD 184, DOW) was prepared using a standard recipe (base:curing agent, 10:1), degassed for 30 min, and poured directly on the negative mold until the mold was full. A secondary degassing was performed for 20 min by placing the PDMS-filled mold in a desiccator under vacuum. To remove excess PDMS, we then covered the PDMS-filled mold with a glass slide to form a flat bottom for the PDMS macrowell insert and cured it at 70°C in an oven for at least 3 h. After that, we removed the glass slide and retrieved the cured PDMS macrowell insert from the mold. We then glued the PDMS macrowell insert to an IVF dish (150255, Thermo Fisher, Waltham, Massachusetts, United States) by pouring a thin layer of uncured PDMS into the base of the IVF dish and curing the assembly for at least 48 h at room temperature. Note that agarose does not bind to PDMS; hence, to prevent the microwell array from floating during culture, an 8-mm biopsy punch (MB5-8, MedBlades, Addison, Illinois, United States) was gently used to remove the PDMS from the base of the macrowells without damaging the IVF dish after curing so that agarose could make contact with the polystyrene dish directly. We also applied a poly-l-lysine coating in the macrowells to enhance the binding as follows: the macrowell dish was first washed in an ultrasonic cleaner with distilled water, dried by a nitrogen spray gun, and sterilized by UV light for at least 1 h. We then loaded 100  μL poly-l-lysine solution (P4707, Sigma Aldrich) in each macrowell, waited for 5 min and rinsed the macrowell with cell culture water (W3500, Sigma Aldrich) three times. The treated dish was air-dried in a biosafety cabinet overnight before use.

#### 2.1.2 Microwell array fabrication (Fig. 5)

Because agarose is soft and difficult to handle, we used a negative mold process to create the agarose microwell array. We designed the microwell mold in SolidWorks. In this design, four cylinders (height: 3 mm, diameter: 6 mm) were placed on a flat base so that the microwell templates could reach the uncured agarose in the macrowell during imprinting. Note that the cylinder diameter is smaller than the macrowell diameter (i.e., 8 mm) so that the cylinders in the mold can easily enter the macrowells to imprint agarose. Each cylinder contained nine identical microwell templates of one of four microwell shapes: truncated conical microwells of two different dimensions, hemispherical microwells, and pyramidal microwells. Although the microwell array included all four designs, in this study, we chose only to evaluate two shapes: the hemispherical microwell shape because it offers optimal embryo culture quality based on research to date,^18^ and the pyramidal microwell shape because it enables spheroid culture and has been widely used in stem cell culture.^35^ Given their flat bottoms, truncated cones do not exhibit some of the aberrations being explored in this work and are thus irrelevant to consider. The disregarded shapes were included in the fabrication protocol out of convenience because we had previously designed a mold that included these shapes.^18^ The dimensions of the explored microwell templates were as follows: hemisphere diameter: 400  μm; and pyramid side length: 400  μm. All templates were 200  μm in height. A drawing of the microwell mold is shown in Fig. S2 in the Supplementary Material. The mold was printed by a 3D printing service (Boston Micro Fabrication) and coated with 2.5 g parylene C to prevent unreacted cytotoxic resin on the mold from entering PDMS and adhesion of the mold to PDMS.

We first weighed agarose and deionized water according to the desired concentration (1%, 1.5% and 2%) and loaded them into a media bottle (1399-125, Corning, New York, United States). Then, we loosely capped the bottle, wrapped the cap with aluminum foil to prevent steam from entering the bottle and affecting the concentration, and sterilized it in an autoclave for 15 min at 121°C. After autoclaving, we transported the bottle to a biosafety cabinet and melted agarose was drawn into a microcentrifuge tube (10025-738, VWR) that was kept in a dry bath at 60°C to prevent the agarose from gelling before usage. We rinsed the surface of the cylinders in the molds with culture media to prevent the formation of an air bubble that could be introduced later in the process when pressing the mold into the agarose in the later step. We then applied 80  μL of agarose to each cylindrical well in the macrowell insert and placed the microwell mold to form impressions in the agarose. To minimize the presence of air bubbles in the melted agarose, we left the dish with the microwell mold in a dry bath for 10 min. After that, we moved the dish from the dry bath to the counter and left the agarose to gel for 20 min at room temperature, after which time we removed the microwell mold.

**Fig. 5:**
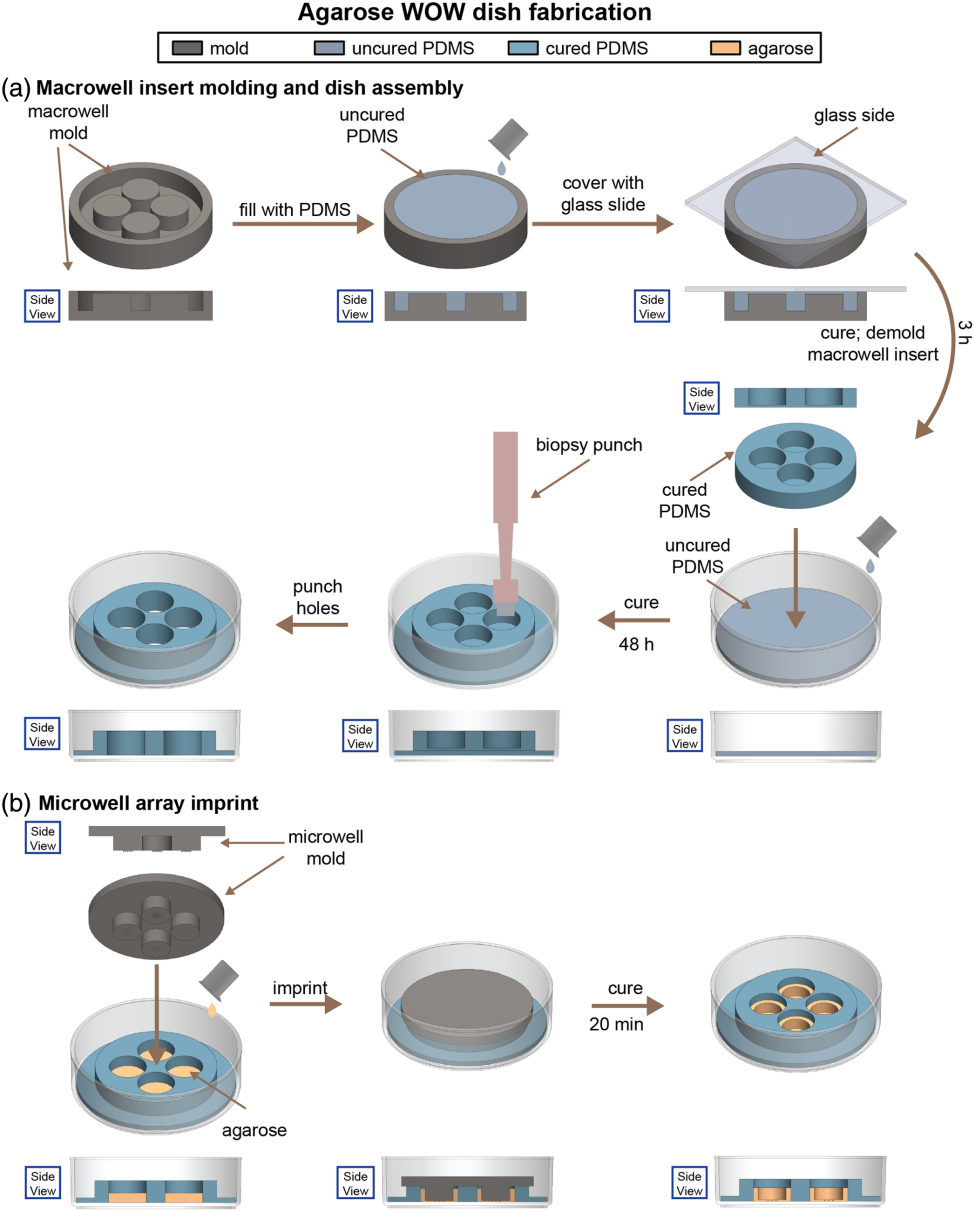
Agarose well-of-the-well (WOW) dish fabrication: (a) fabrication of the PDMS macrowell insert and assembly of a macrowell insert in an IVF dish; (b) fabrication of the agarose microwell array. Note: not drawn to scale.

### 2.2 PDMS WOW Dish Fabrication

**Fig. 6:**
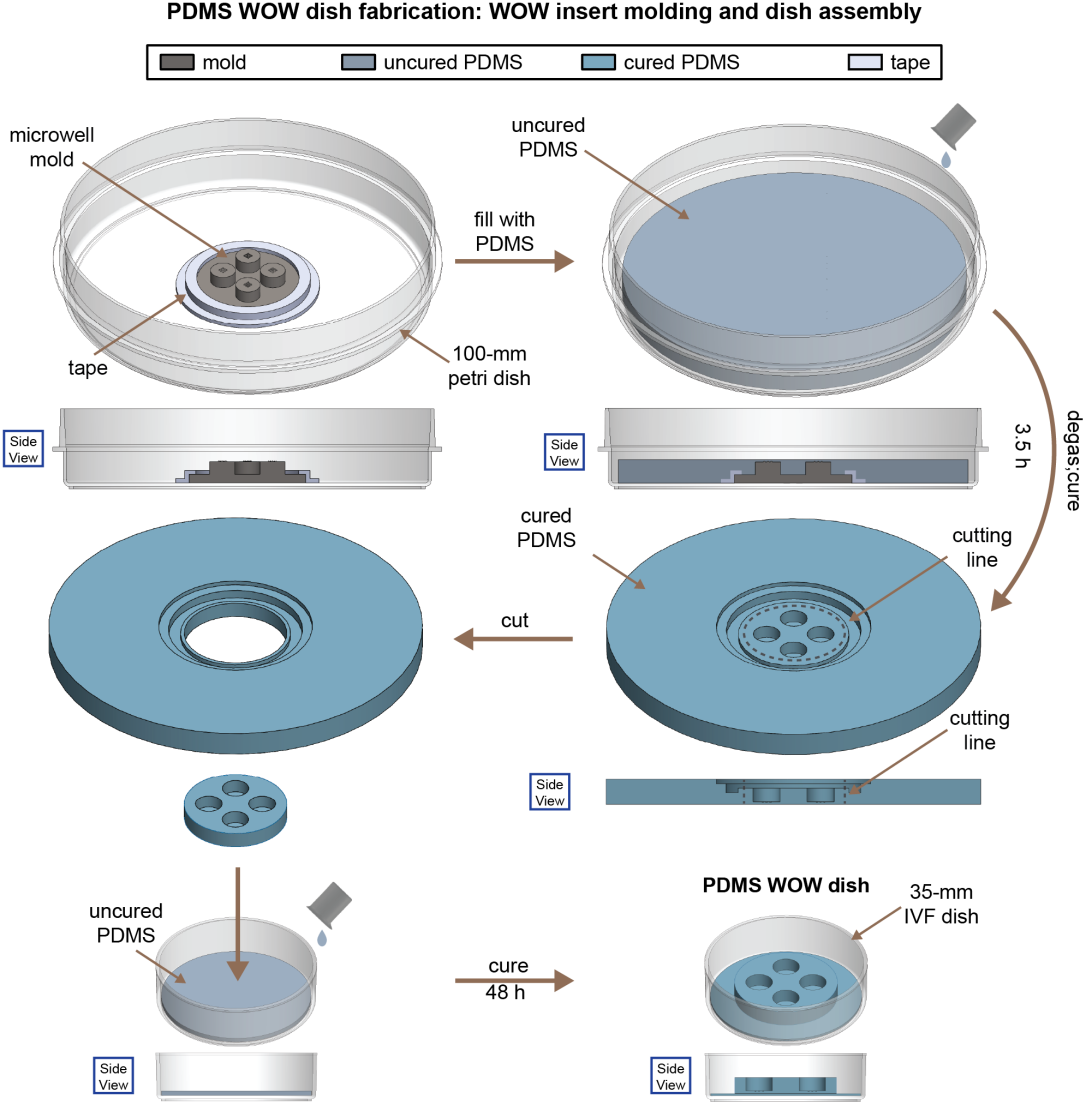
Polydimethylsiloxane (PDMS) well-of-the-well (WOW) dish fabrication. Note: not drawn to scale; tape shape is illustrative and not representative of true shape.

We fabricated high-RI WOW dishes comprising PDMS macrowells and microwells to compare the effects of RI on distortion and resolution with low-RI agarose WOW dishes. The fabrication process is shown in Fig. 6 and differs from Fig. 5 in that the macrowell and microwells were created in the same step from a single mold. The mold used was the same mold previously applied to produce the agarose microwell array and was taped (scotch tape, 3M) at its base to a 100-mm petri dish (752401, NEST Scientific, Woodbridge, New Jersey, United States). PDMS was prepared the same way as for macrowell fabrication and was poured into a petri dish until it covered the mold. A secondary degassing was performed for 20 min by placing the petri dish in a desiccator under vacuum to remove the air bubble introduced when pouring the PDMS. Then, we placed the petri dish in an oven at 70°C for at least 3 h to cure the PDMS. After curing, we demolded the PDMS insert and cut it to fit a 35-mm IVF dish with a razor blade. The insert was glued to an IVF dish by pouring a layer of uncured PDMS into the base of the IVF dish. The whole assembly was left to cure at room temperature for at least two days. Finally, the dish with the macrowell insert was washed in an ultrasonic cleaner with distilled water, dried by a nitrogen spray gun, and sterilized by UV light for at least 1 h.

### 2.3 Agarose WOW Dish Inspection and Characterization

#### 2.3.1 Microwell array inspection

To confirm the integrity of the microwell structures in the WOW dish, we imaged the microwell array using optical coherence tomography (OCT, TEL221PSC1, Thorlabs, Newton, New Jersey, United States), a 3D imaging modality that can visualize the cross-sectional depth profile of microwells. For testing purposes, we only inspected the WOW dish containing a 2%-agarose microwell array as this concentration provides the best visibility due to the strong refractive index mismatch with water. The imaging was performed over two 2×2  mm scanning areas that covered the hemispherical and pyramidal microwell arrays separately. The acquired volumetric images were then loaded into 3D Slicer (Harvard Medical School) and ImageJ (NIH) for processing. Average intensity projections along the z-axis were generated to characterize the dimensions of lateral (i.e., x-y plane) structures. We also extracted cross-sectional images to visualize the profiles of the microwell structures. From these, we measured the average diameters and depths of hemispherical microwells and the average side lengths and depths of pyramidal microwells. Note that OCT measures optical path difference instead of physical distance. The measurement results were converted to physical distance by using the RI of water at the central wavelength (1300 nm) of OCT (i.e., RI = 1.32^36^).

#### 2.3.2 Resolution assessment

**Fig. 7:**
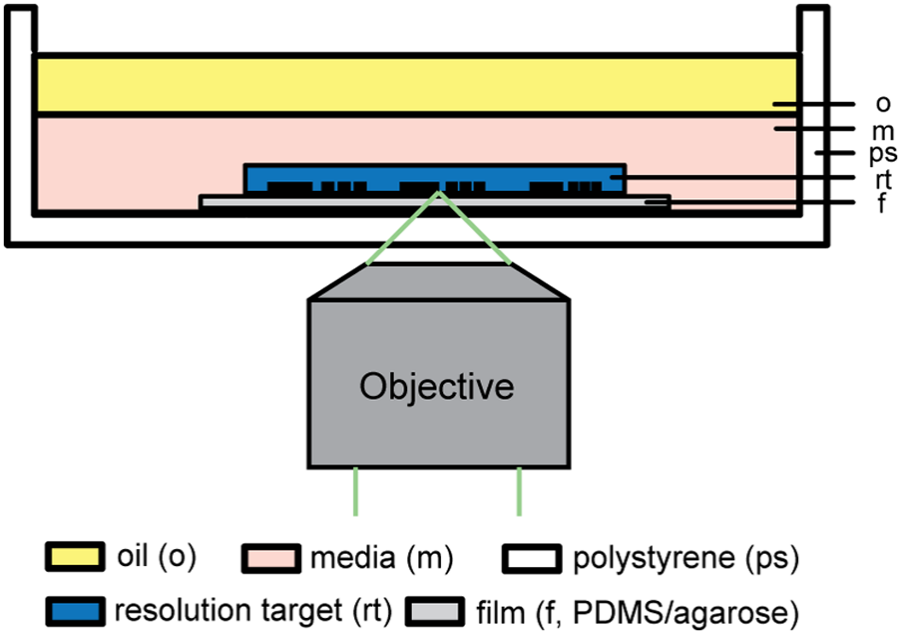
Optical system configuration for resolution assessment. Note: not drawn to scale.

Due to the limited size of the microwell, it is impractical to measure the resolution inside the microwell using a standard resolution target. Alternatively, to assess the optical resolution of imaging through substrates with different RIs, we fabricated films comprising agarose of different concentrations or PDMS and applied the ISO 12233 slant edge method to measure the line spread function. Details of the slant edge method can be found in other references.^37^^,^^38^ To fabricate agarose films, we first weighed 100, 150, and 200 mg of agarose (16500100, Thermo Fisher) in 10 mL distilled water, leading to 1%, 1.5% and 2% agarose. Then, we melted the agarose in an autoclave the same way as described for microwell array fabrication. Melted agarose was loaded into a syringe. We placed a ring shim (0.0125 inch in thickness, 98090A971, McMaster Carr) on a glass slide (71860-01, Electron Microscopy Scientific), applied sufficient agarose from the syringe to fill the shim, and pressed another glass slide onto the shim. The assembly was left at room temperature to gel for 20 min. After that, we removed the top glass side and carefully loaded the film into an IVF dish. To fabricate the PDMS film, we first prepared the PDMS in the same way as for macrowell fabrication. We placed the same ring shim used for the agarose film on a glass slide and applied sufficient PDMS to fill the shim. A second degassing was performed by leaving the PDMS in a desiccator under vacuum for 20 min to remove air bubbles introduced from pouring the PDMS. After that, we pressed the bottom of a 100-mm petri dish onto the shim. A petri dish was used instead of a glass side to facilitate the retrieval of the PDMS film. The assembly was placed in an oven at 70°C for at least 3 h to cure the PDMS. The cured PDMS film was then loaded into an IVF dish. For imaging, we placed a United States Air Force (USAF) resolution target (R1L1S1P, Thorlabs) with the bars facing down toward the objective on top of the agarose and PDMS films. We then pipetted 3 mL of embryo culture media (MR-101, Sigma Aldrich) to cover the top of the target, followed by 2 mL of embryo culture oil (9305, Fujifilm Scientific, Santa Ana, California, United States). The configuration of the prepared dish is shown in Fig. 7. The prepared dish was loaded in a stage-top incubator with 6% ${\mathrm{CO}}_{2}$ at 37°C on an inverted microscope (DMI8, Leica, Wetzlar, Germany) and incubated for at least 30 min.

Prior to imaging, the dish was rotated until the bottom edge of the square bar on the USAF target was ∼30  deg counterclockwise relative to the horizontal axis of the camera. We imaged the right edge of the square under BF mode on a microscope with a 20× long working distance objective (378-824-16, Mitutoyo, Kawasaki, Japan). Ten images were taken for each condition. We then measured the line spread function on the images in ImageJ with the Slanted Edge MTF plug-in. The full width half maximum of the line spread function was considered the resolution. We averaged the 10 resolution measurements from each film to reduce the errors.

### 2.4 Fabrication Reproducibility Test

To evaluate the reproducibility of the proposed fabrication method, we made three agarose WOW dishes in succession and compared the microwell structures by characterization with OCT. We first followed the proposed fabrication process and made three agarose WOW dishes using the same mold. A concentration of 1.5% (w/v%) of agarose was used in this study as it was shown to be the optimal concentration for RI matching. The macrowells were filled with deionized water for imaging. Then, we imaged the microwell array using OCT. The imaging was performed over two 2×2  mm scanning areas that covered the hemispherical and pyramidal microwell arrays separately, which is the same as in the initial characterization. The acquired volumetric images were then loaded into 3D Slicer and ImageJ for processing. Average intensity projections along the z-axis were generated to characterize the dimensions of lateral (i.e., x–y plane) structures. We also extracted cross-sectional images to visualize the profiles of the microwell structures. From these, we measured the average diameters and depths of hemispherical microwells and the average side lengths and depths of pyramidal microwells.

### 2.5 Geometric Distortion Assessment on WOW Dishes of Different RIs

Due to the small dimensions of the microwells, assessing distortion using a standard grid chart target was not practical. Alternatively, we followed a similar protocol that has been used to measure and calibrate the distortion of a confocal microscope.^39^ We used microspheres with a known diameter and assessed distortion by measuring the diameters of microspheres placed in the microwells. Certified microspheres (4310A, Thermo Fisher) with a diameter of 99  μm, which is close to the diameter of a pre-implantation embryo, were used in the experiment. Three types of culture dishes were applied in the study: an IVF dish with a flat substrate for the baseline measurement, a PDMS WOW dish (high-RI), and an agarose WOW dish (low-RI). Nine measurements were performed in each condition.

#### 2.5.1 Flat dish preparation

To acquire the baseline microsphere diameter on a flat substrate, we placed nine microspheres by micropipette (7-72-2170/1, Cooper Surgical, Trumbull, Connecticut, United States) in an 80  μL droplet of embryo culture media covered with 3 mL of embryo culture oil in an IVF dish.

#### 2.5.2 PDMS WOW dish preparation

Due to the hydrophobicity of PDMS, bubbles may be present in microwells when pipetting culture media in macrowells. To reduce bubbles, PDMS WOW dishes were pre-warmed in an incubator overnight at 37°C. We then pipetted 80  μL of pre-warmed culture media into the macrowells. After loading the media, the PDMS WOW dishes were inspected for bubbles under a stereomicroscope, and bubbles were carefully removed by micropipette if observed. We then used a micropipette to load one microsphere in each of the nine hemispherical microwells and pyramidal microwells. After loading, we covered the culture media with 2.5 mL culture oil.

#### 2.5.3 Agarose WOW dish preparation

To mimic embryo imaging conditions, we rinsed the agarose inserts by pipetting 80  μL embryo culture media into the macrowells and incubating the dish for 10 min at room temperature. Then, we removed the culture media and loaded one microsphere in each of the nine hemispherical microwells and pyramidal microwells. Finally, we pipetted 80  μL of embryo culture media into the macrowells, covered with 2.5 mL culture oil.

#### 2.5.4 Microscopy imaging

Each of the aforementioned prepared dishes was loaded into a stage-top incubator with 6% ${\mathrm{CO}}_{2}$ at 37°C on an inverted microscope and incubated for at least 30 min. A stage-top incubator was employed to mimic the live-cell imaging condition of embryos in IVF clinics; more importantly, the stage-top incubator maintained a consistent temperature across all measurements as temperature is a known factor that changes the RI. Following temperature acclimation, we imaged the microspheres under BF mode of the microscope with a 20× long working distance objective; its focus was carefully adjusted to match the edge of the microsphere to minimize the effects of blurring on the diameter measurement.

#### 2.5.5 Diameter measurement

Collected images were loaded in MATLAB (MathWorks, Natick, Massachusetts, United States), and the microspheres were automatically segmented with the Image Segmenter Toolbox (MathWorks). The centroid was first determined by averaging the x- and y-pixel coordinates of all points on the segmented boundaries. We then calculated radii by measuring the distance from the centroid to each boundary point. From these measurements, we determined the mean radius and standard deviation for each microsphere, which we then converted to and reported as diameters. Using the known pixel-to-distance ratio (i.e., 3.2  pixel/μm) calibrated by a ruler target, the diameters and diameter standard deviations of the microspheres were calculated. We then performed statistical analysis on the data. We first verified the normal distribution of each experimental group by the Shapiro–Wilk normality test. We found the diameters of microspheres follow a normal distribution; thus, a Tukey’s one-way ANOVA analysis was performed between experimental groups for hemispherical and pyramidal microwells individually. We found the standard deviations of the microsphere diameters do not follow a normal distribution; thus, a nonparametric Kruskal–Wallis’s ANOVA was performed between experimental groups for hemispherical and pyramidal microwells individually. All statistical analyses were performed in OriginPro (OriginLab, Northampton, Massachusetts, United States).

### 2.6 Geometric Distortion Assessment on WOW Dishes of Different Roughness

To investigate the effect of 3D-printed ridges independent of material on geometric distortion, we performed imaging in a PDMS WOW dish with the same microwell design as the currently proposed dish, but its mold was produced by a lower resolution 3D printer, leading to higher surface roughness than the molds produced in the current work. The fabrication process is documented in Ref. 18.

#### 2.6.1 Rough PDMS WOW dish preparation

The rough PDMS WOW dish was prepared the same way as the current PDMS WOW dishes. In brief, each macrowell was filled with 80  μL culture media, and one microsphere was loaded in each of the nine hemispherical microwells and pyramidal microwells. The culture media were covered with 2.5 mL culture oil.

#### 2.6.2 Microscopy imaging

The rough PDMS WOW dish was loaded in a stage-top incubator with 6% ${\mathrm{CO}}_{2}$ at 37°C on an inverted microscope and incubated for at least 30 min. We then imaged the microspheres under the BF mode of the microscope with a 20× long-working-distance objective whose focus was carefully adjusted to match the edge of the microsphere to minimize the effects of blurring on the diameter measurement.

#### 2.6.3 Diameter measurement

Collected images were loaded in MATLAB (MathWorks), and the microspheres were automatically segmented with the Image Segmenter Toolbox (MathWorks). Using the known pixel-to-distance ratio (i.e., 3.2  pixel/μm) calibrated by a ruler target, the diameters and diameter standard deviations of the microspheres were calculated. We then performed statistical analysis on the data of the rough PDMS WOW dish and compared it with the data for the PDMS WOW and IVF dish acquired from the previous experiment. We tested for a normal distribution in each experimental group by the Shapiro–Wilk normality test. We found that the diameters and standard deviations of the microspheres for the pyramidal microwell did not follow a normal distribution; thus, a nonparametric Kruskal–Wallis’s ANOVA was performed between experimental groups. All statistical analyses were performed in OriginPro (OriginLab).

### 2.7 Optical Aberrations Assessment on WOW Dishes of Different RIs

To directly measure optical aberrations introduced by WOW dishes of different RIs, we added a wavefront sensor to the microscope and analyzed the Zernike polynomials to characterize different aberrations.

A Shack–Hartmann wavefront sensor (WFS40-7AR, Thorlabs) was integrated into the inverted microscope through the documentation port to enable direct pupil-plane wavefront acquisition. An achromatic lens (AC254-200-A, Thorlabs) with a 200 mm focal length—matching the microscope’s built-in tube lens—was added after the documentation port. This lens paired with the tube lens to form a relay system that projected the objective’s back aperture onto the wavefront sensor. The relay lens and Shack–Hartmann sensor were positioned so that the objective pupil was sharply imaged and uniformly filled on the sensor array. To minimize chromatic blur at the microlens array, a red color filter (Red 43R25, Tiffen, Hauppauge, New York, United States) was placed after the field lens to narrow the illumination spectrum of the LED light source.

During wavefront acquisition, the condenser aperture diaphragm was closed to reduce the illumination angle and suppress aberrations introduced by oblique illumination geometry. The field diaphragm was also stopped down so that the illuminated area on the sample matched the size of a single microwell, thereby restricting aberration sampling to the region of interest. We fabricated a PDMS WOW dish and agarose WOW dishes at 2%, 1.5%, and 1% concentrations following the methods described above. We also prepared an IVF dish containing 80  μL culture media and 3 mL culture oil to obtain a baseline measurement. For WOW dishes, we first focused on the base of the microwells using the eyepiece. We then switched to the documentation port and adjusted the Köhler illumination by maximizing the sharpness of the field diaphragm edge. Light intensity was adjusted to avoid saturation of the wavefront sensor. Nine measurements were performed for each condition. For the IVF dish, we first focused on the boundary between culture media and oil using the eyepiece. We then switched to the documentation port. During the measurement, we found that the rounded shape of the culture media could affect the wavefront shape when imaging the droplet edge. To avoid this and obtain a baseline measurement, we carefully moved the stage to position the central area in the field of view until a circular wavefront was observed. After that, we adjusted the Köhler illumination by maximizing the sharpness of the field diaphragm edge and light intensity to avoid saturation of the wavefront sensor. Nine measurements were conducted on the IVF dish condition. All wavefront data were collected and processed using the Shack–Hartmann Wavefront Sensor Software Package (Thorlabs). The pupil size was set to 5.1 mm to cover the objective’s back aperture wavefront. Reconstructed phase maps were decomposed into Zernike polynomials up to the fifth radial order. We extracted the Zernike coefficients and reported the root-mean-square (RMS) variation for each aberration order. The zero and first orders of Zernike polynomials—indicating position and tilt that do not affect imaging quality—were excluded from subsequent analysis.

After the measurement, we performed statistical analysis on the RMS variations. We first verified the normal distribution of each experimental group using the Shapiro–Wilk normality test and found that some groups did not follow a normal distribution. Therefore, a nonparametric Kruskal–Wallis ANOVA was performed between experimental groups for hemispherical and pyramidal microwells individually at each aberration order. All statistical analyses were performed in OriginPro (OriginLab).

### 2.8 Agarose Stability Test

To assess the stability and integrity of agarose microwells after 6-day culture in an incubator, including 2 days of agarose pre-culture and four days of embryo culture, we conducted a stability test. Agarose WOW dishes of 1%, 1.5%, and 2% (w/v%) concentration were made following the proposed fabrication process. We then filled each macrowell with 80  μL of embryo culture media and covered them with 2.5 mL of embryo culture oil. The dishes were kept in an incubator at 6% ${\mathrm{CO}}_{2}$ and 37°C for 6 days. After the incubation, we collected the dish, aspirated the embryo culture media and oil, and rinsed the dish with deionized water three times. This step was to eliminate the interruptions of oil for later imaging. The macrowells were then filled with deionized water for imaging. We imaged the microwell array using OCT. The imaging was performed over two 2  mm×2  mm scanning areas that covered the hemispherical and pyramidal microwell arrays separately, which is the same as the initial characterization. The acquired volumetric images were then loaded into 3D Slicer and ImageJ for processing. Average intensity projections along the z-axis were generated to characterize the dimensions of lateral (i.e., x–y plane) structures. We also extracted cross-sectional images to visualize the profiles of the microwell structures. From these, we measured the average diameters and depths of hemispherical microwells and the average side lengths and depths of pyramidal microwells.

### 2.9 Mouse Embryo Culture and Imaging

To demonstrate the utility of *in situ* imaging in the agarose WOW dish, we cultured one-cell mouse embryos (B6C3F1 × B6D2F1, Embryotech Laboratories, Haverhill, Massachusetts, United States) and imaged the blastocysts that resulted after four days. In this experiment, we used 1.5% agarose because our test results revealed that this recipe best balances the performance of optical resolution and geometric distortion (see Sec. 3).

#### 2.9.1 Agarose WOW dish preparation

On day 1, the same protocol as described in the agarose WOW dish fabrication section was applied to make an agarose WOW dish. In this experiment, cell culture-grade water instead of distilled water was used to fabricate the agarose microwell array to reduce the risk of water osmolarity impacting the embryo growth. In addition, we found that the water content in the fabricated agarose microwell array can dilute the culture media and affect the embryo growth. To minimize this risk, before usage, the fabricated agarose WOW dish was incubated with 3 mL culture media in a water-jacketed ${\mathrm{CO}}_{2}$ incubator at 37°C under 6% ${\mathrm{CO}}_{2}$ for two nights with one media change on day 2.

#### 2.9.2 Embryo thawing

On day 3, cryopreserved one-cell mouse embryos were thawed by following the instructions from the vendor. We exposed a straw of 10 embryos to room temperature for 2 min and immersed the straw in a 37°C water bath for 1 min. We then removed the straw from the water bath and wiped it dry with a tissue. We cut the straw at the plug and seal and pushed all of its contents into a droplet of 250  μL of embryo culture media that had been pre-warmed in an IVF dish (150255, Thermo Fisher). After that, we immediately rinsed the embryos twice in two droplets of 250  μL of pre-warmed culture media in IVF dishes set aside for rinsing. The embryos were left in the second rinsing droplet and incubated for 10 min at 37°C under 6% ${\mathrm{CO}}_{2}$ to recover.

#### 2.9.3 Embryo culture

On day 2, we preequilibrated 1 mL embryo culture media in a tube (5000-1020, Thermo Fisher) with a loosened cap and prewarmed 3 mL embryo culture oil in the incubator at 37°C under 6% ${\mathrm{CO}}_{2}$ overnight. On day 3, after the embryos were recovered, we drained the culture media in the prepared agarose WOW dish to visualize the microwells. The embryos were then seeded in the hemispherical and pyramidal microwells using micropipettes under a stereomicroscope with an attached, 37°C warm plate. We then pipetted 80  μL preequilibrated culture media into macrowells and covered them with 2.5 mL of prewarmed embryo culture oil. The embryos were cultured in the incubator at 37°C under 6% ${\mathrm{CO}}_{2}$ for 4 days.

#### 2.9.4 Embryo imaging

After 4 days of culture, the agarose WOW dish was directly loaded into a stage-top incubator at 37°C under 6% ${\mathrm{CO}}_{2}$ and imaged on an inverted microscope using a 20× objective under regular BF mode.

## 3 Results and Discussion

### 3.1 Microwell Array Characterization

**Fig. 8:**
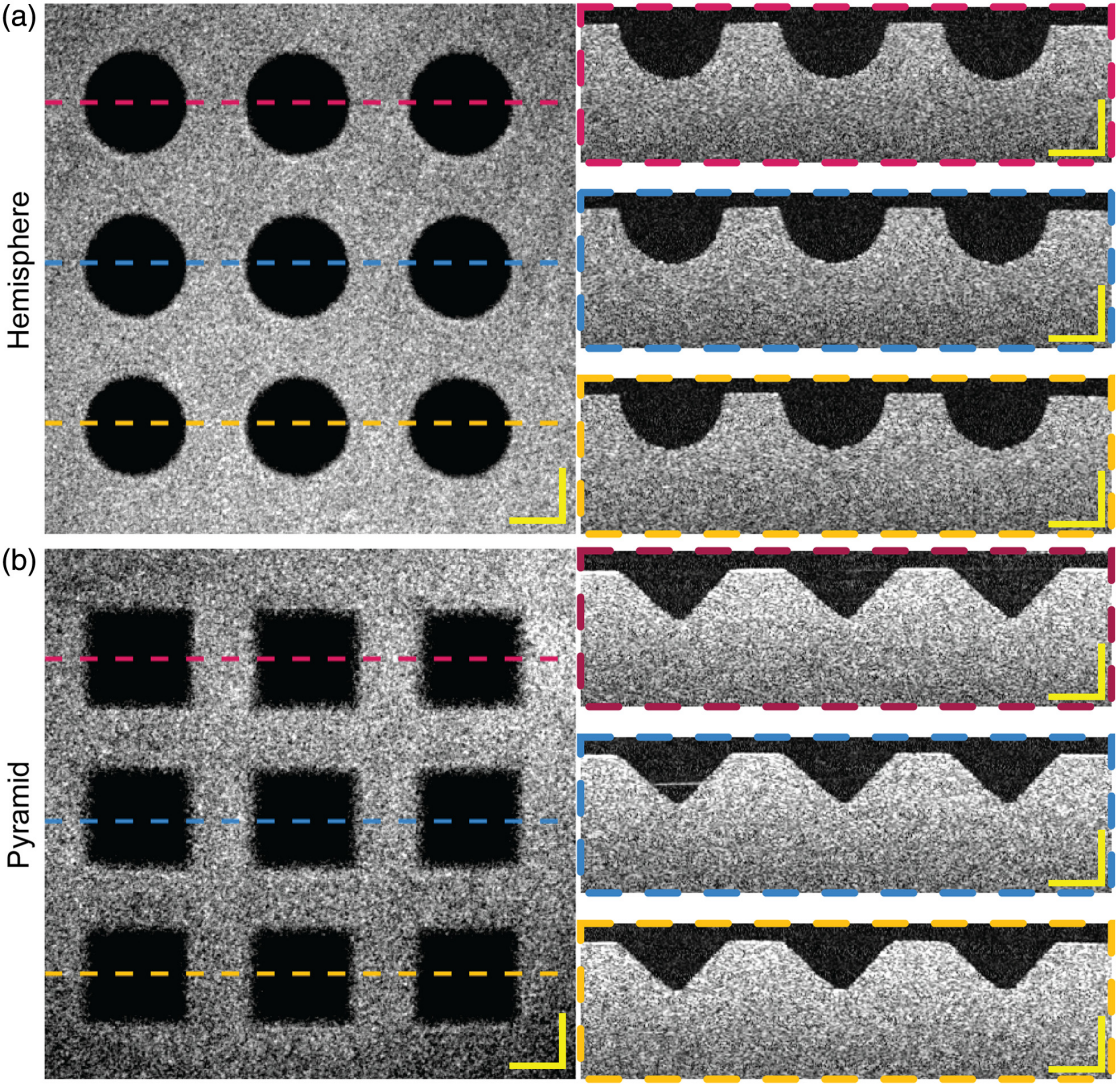
OCT images of the agarose microwell array. Images of the (a) hemispherical microwell array and (b) pyramidal microwell array, including averaged intensity z-projections and cross-sectional images (scale bar: 200  μm), show high-fidelity transfer of intended structures from the mold to the array.

En face and side view images of fabricated microwells inspected by OCT are shown in Fig. 8, where the top row shows the hemispherical microwells and the bottom row shows the pyramidal microwells. Table 1 summarizes the observed dimensions. The measured dimensions are slightly off from the design but are within the tolerance of the 3D printer and materials (±25  μm) used to produce the mold. All microstructures were reliably transferred from the mold and met design requirements, confirming the reliability of our fabrication process.

**Table 1:** Dimension characterization of hemispherical and pyramidal microwells in an agarose microwell array (number of microwells, n=9, SEM, standard error of mean).

	Hemispherical microwell	Design	Pyramidal microwell
Diameter/side length (mean ± SEM, mm)	390.8 ± 2.3	400	396.8 ± 1.3
Depth (mean ± SEM, mm)	200.7 ± 0.8	200	186.9 ± 0.9

### 3.2 Reproducibility Test

**Fig. 9:**
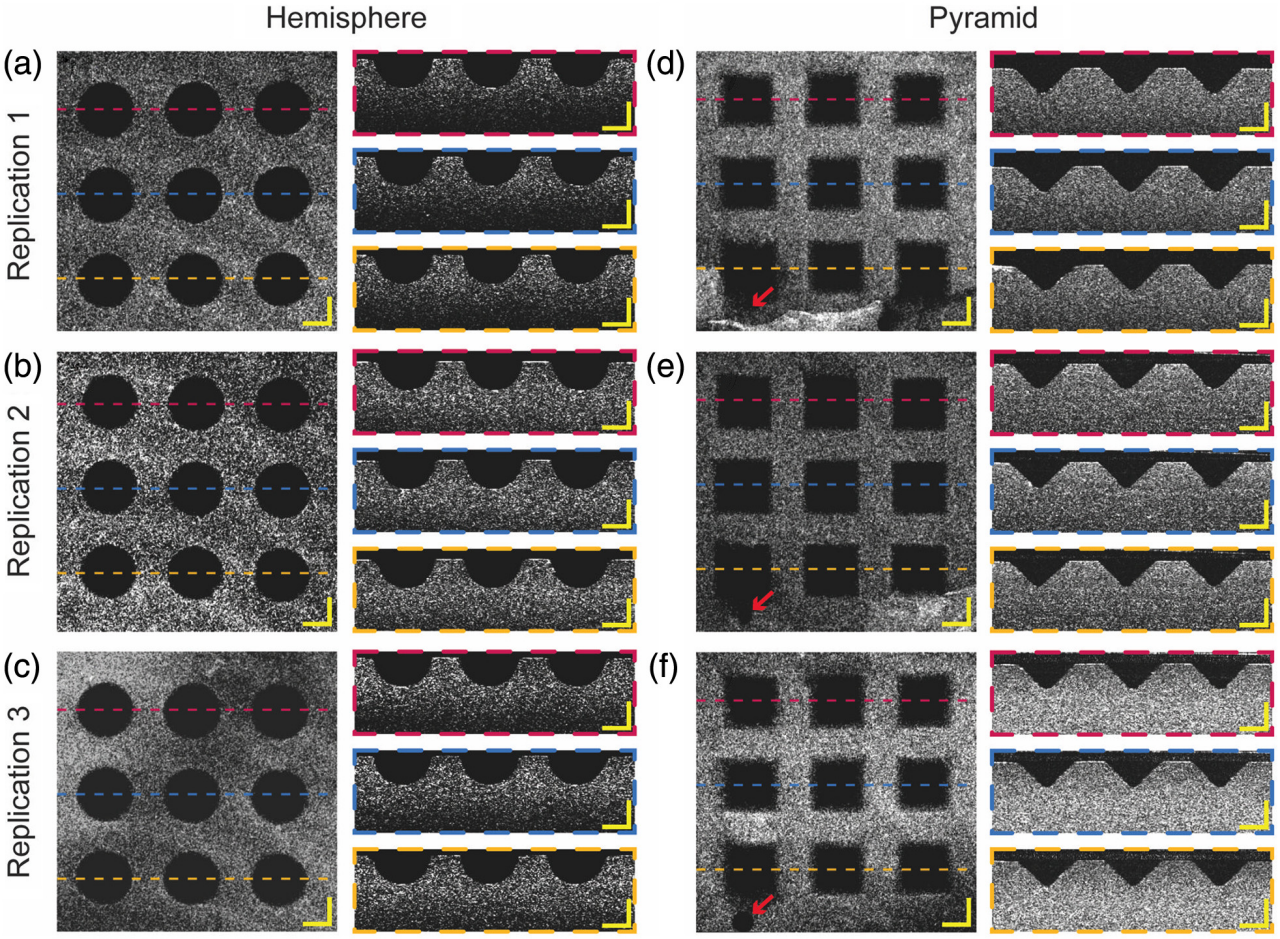
OCT images of the three replications of the agarose microwell array. Images of the (a)–(c) hemispherical microwell array and (d)–(f) pyramidal microwell array, including averaged intensity z-projections and cross-sectional images (scale bar: 200  μm). The breakage of the microwell and potential presence of an air bubble during fabrication are pointed out by the red arrow.

**Fig. 10:**
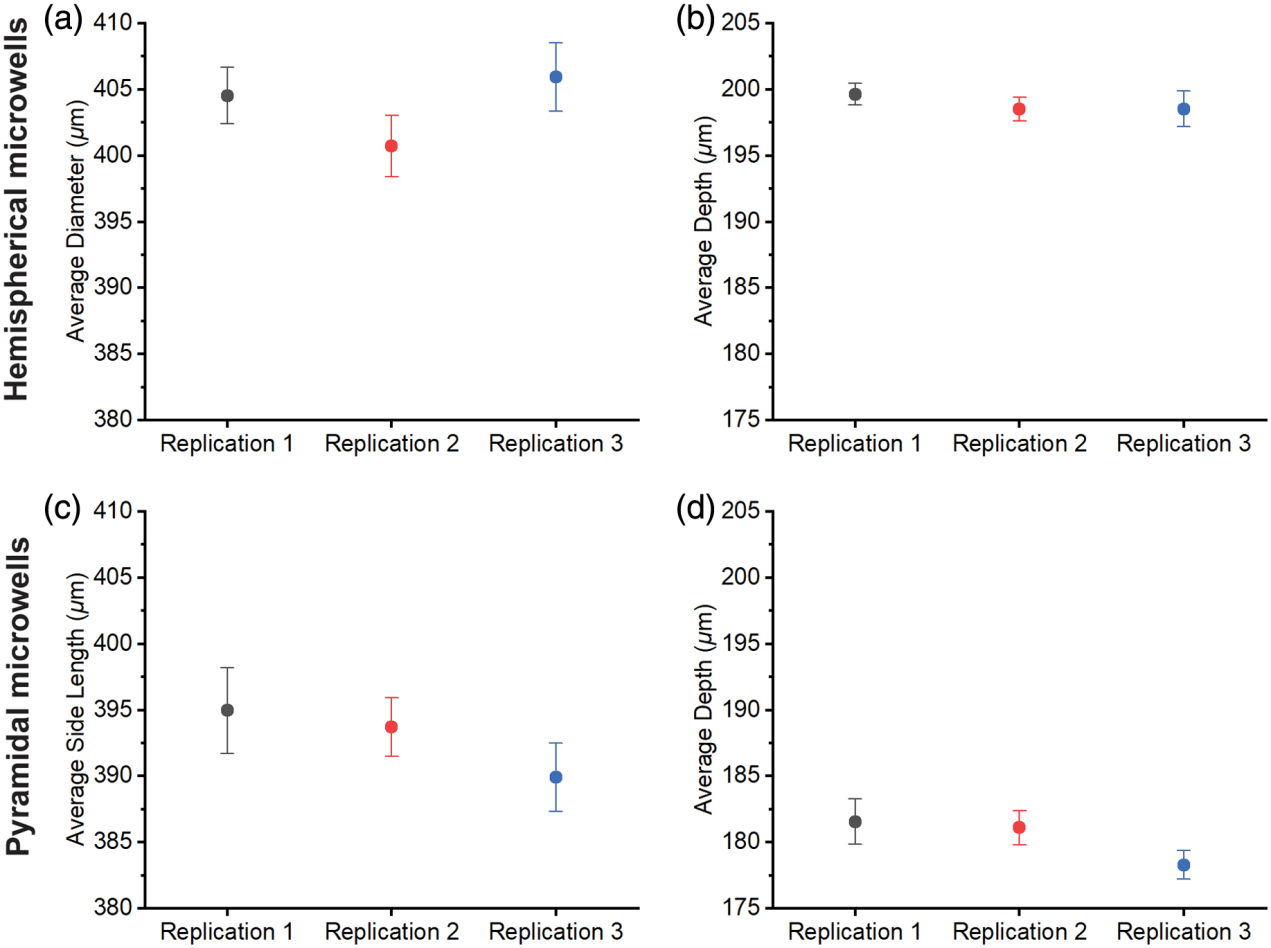
Comparisons of average diameter (a) and depth (b) for the hemispherical microwell and average side length (c) and depth (d) for the pyramidal microwell. The bar indicates the standard error of the mean.

En face and side view images of the fabricated microwells inspected by OCT across three replications are shown in Fig. 9, where the left and right columns show the hemispherical and pyramidal microwells, respectively, and each row shows one replication. Quantitative comparisons of the diameter and depth for the hemispherical microwell and side length and depth for the pyramidal microwell are summarized in Fig. 10. Although we observed the presence of air bubbles and breakage at the openings of one pyramidal microwell, marked by red arrows in the en face images, all the microwells retain their shapes across three replications, as shown in the side view images in Fig. 9. Note that the defects at the opening of microwells should not affect the usage. Quantitatively, the maximum difference between replications of measurements is less than 6  μm, indicating acceptable reproducibility.

### 3.3 Resolution Assessment of PDMS and Agarose

**Fig. 11:**
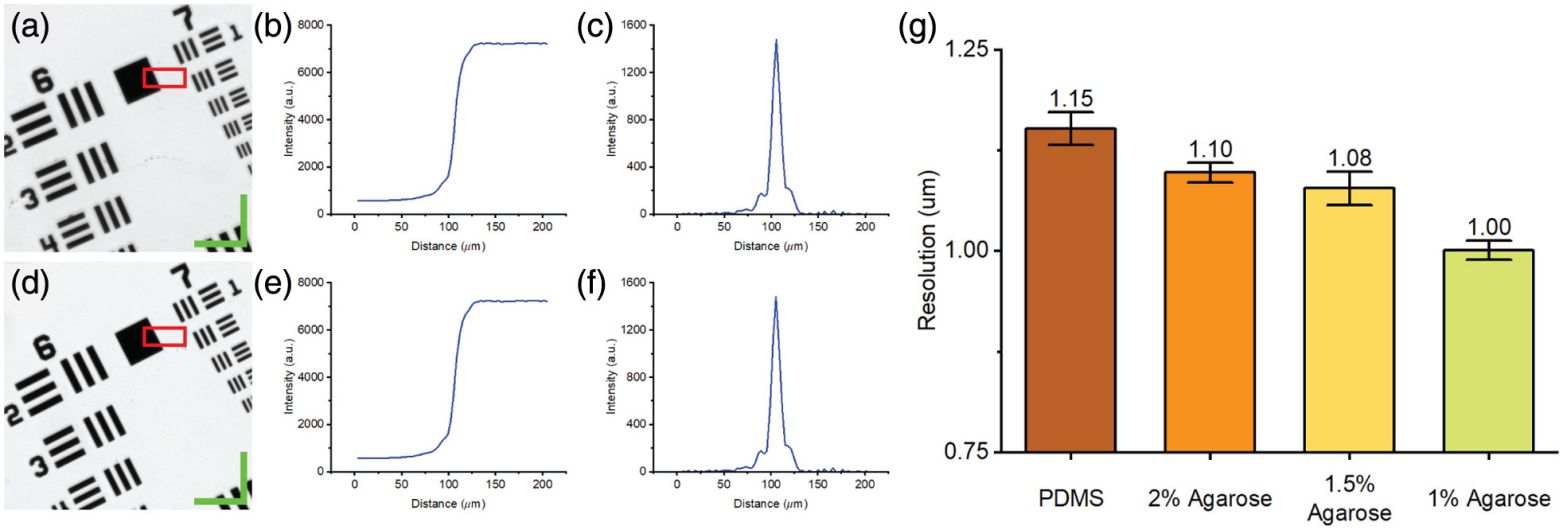
Example images from the resolution measurement using the slant edge method on PDMS (top row) and 1% agarose (bottom row) and measurement results: (a), (d) USAF resolution target image (scale bar: 50  μm), (b), (e) edge response function, (c), (f) line spread function, and (g) resolution comparison of PDMS and agarose of different concentrations (n=10). The area for measurement is labeled by the red box.

Figure 11 shows the results of the resolution assessment using the slant edge method on PDMS and agarose films. Figures 11(a) and 11(d) show images of a USAF target on a PDMS film and 1% agarose film, respectively, wherein the area for resolution measurement is labeled by a red box. The edge response functions extracted from the slant edge of the boxed area of PDMS and 1% agarose are shown in Figs. 11(b) and 11(e). Differentiation of the edge response functions yields the line spread functions for PDMS and 1% agarose, shown in Figs. 11(c) and 11(f). Figure 11(g) shows the resolutions of PDMS and agarose of different concentrations. We found that the resolution improves with the reduction of the RI and that agarose, in general, offered better resolution than PDMS. Overall, 1% agarose shows the best resolution among all tested conditions.

### 3.4 Geometric Distortion Assessment on the WOW Dishes of Different RIs

**Fig. 12:**
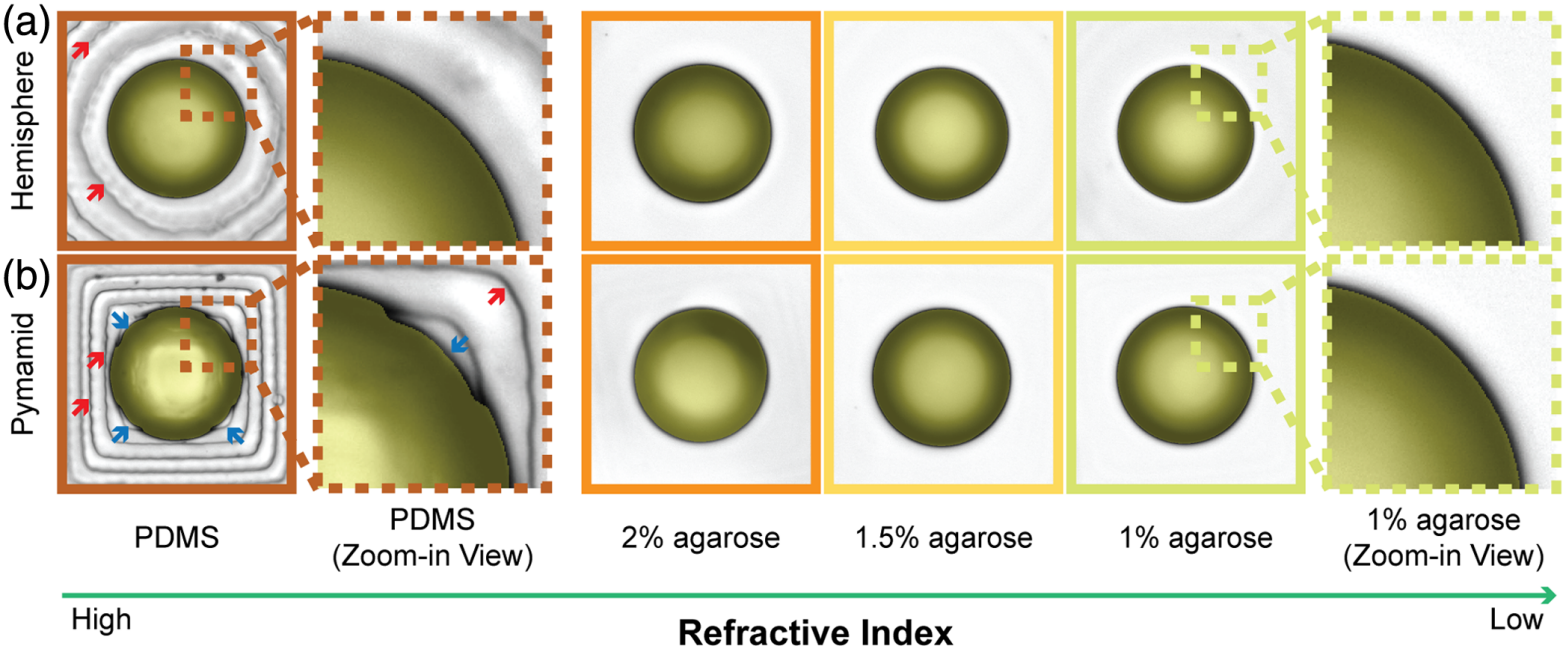
Representative microsphere images in hemispherical (a) and pyramidal (b) microwells of PDMS and agarose of different concentrations. The ridges introduced by the 3D-printed mold are indicated by red arrows. The deformations introduced by the shape of pyramidal microwells are indicated by blue arrows. The field of view of each bead image is 160×160  μm.

Figure 12 shows representative images of microspheres in PDMS and agarose microwells. The microsphere is highlighted as a yellow overlay in the image. Notably, the layer-by-layer ridges seen in the PDMS microwells caused by the 3D printing, indicated by the red arrows in Fig. 12, are strongly visible in the PDMS microwell but can barely be seen in the agarose microwells. This shows that our RI matching strategy works to reduce optical aberrations. Careful inspection of the pyramidal PDMS microwell also shows geometric distortions visible for PDMS microwells, indicated by the blue arrows in Fig. 12, where the microsphere appears visibly deformed at the four corners due to the edges of the pyramidal microwell. These distortions are similarly absent in the index-matched agarose microwells.

#### 3.4.1 Diameter measurements

**Fig. 13:**
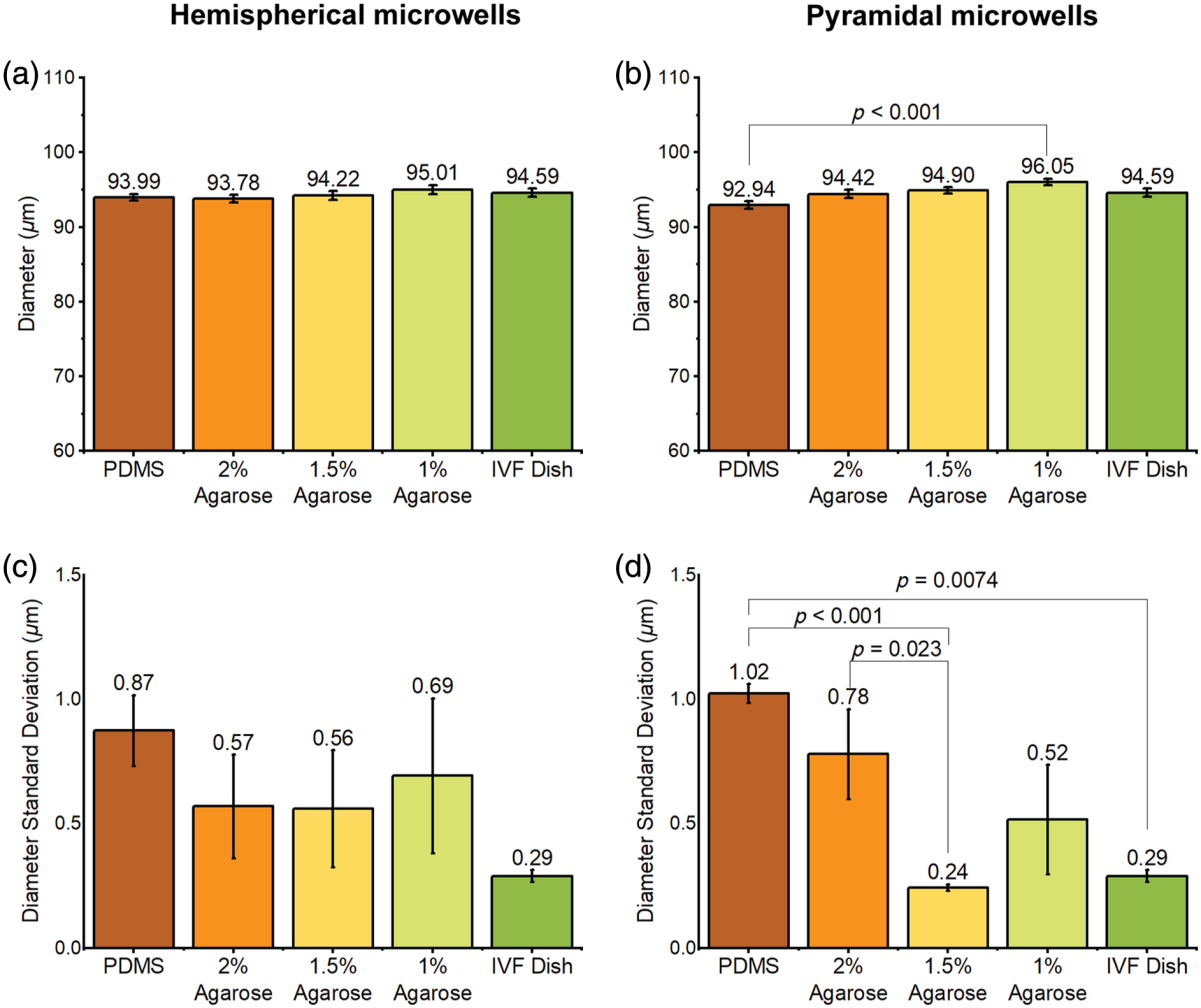
Microsphere diameter and diameter standard deviation measurements in (a), (b) hemispherical and (c), (d) pyramidal microwells (n=9).

To assess the level of distortion, we compared the microsphere diameters and their standard deviations when placed in different culture dishes. The results are summarized in Fig. 13. For the hemispherical microwells [Figs. 13(a) and 13(c)], the diameters of microspheres in 1.5% agarose microwells are more similar to those in the IVF dish than those in PDMS, 2% and 1% agarose dishes; however, the diameters are similar among all testing groups, and no significant difference was found in any paired group when tested by Tukey’s one-way ANOVA. The standard deviations of the microsphere diameters in agarose are lower than those in PDMS, with 1.5% agarose exhibiting the lowest standard deviations among all testing groups except the IVF dish condition, indicating a more uniformly circular appearance. However, no significant difference was found in any paired group when tested by Kruskal–Wallis’s one-way ANOVA.

In pyramidal microwells, the diameters [Fig. 13(b)] of microspheres in 2% agarose match better to the IVF dish condition than do PDMS, 1% and 1.5% agarose. A statistically significant difference was found between 1% agarose and PDMS when tested by Tukey’s one-way ANOVA. Overall, the diameters are similar among all testing groups. The diameter standard deviations [Fig. 13(d)] in agarose are lower than in PDMS, and 1.5% agarose shows the lowest standard deviations among agarose groups. A significant difference in the standard deviations of microsphere diameters was found between PDMS and 1.5% agarose, PDMS and IVF dishes, and between 2% and 1.5% agarose dishes, whereas no significant difference was found between all agarose and IVF dishes when tested by Kruskal–Wallis’s one-way ANOVA. These results indicate that a more uniformly circular microsphere appearance can be achieved with some agarose concentrations than with PDMS, resulting in less geometric distortion.

Overall, these results suggest that microsphere diameters measured in 1.5% agarose better match the ground truth diameters measured in IVF dishes and provide lower diameter variations. Moreover, WOW dishes with microwells made of 1.5% agarose may provide less geometric distortion than PDMS, 2% and 1% agarose. To balance the performance of optical resolution and geometric distortion, we believe that 1.5% agarose is optimal among all testing conditions.

### 3.5 Geometric Distortion Assessment on the WOW Dishes of Different Roughness

**Fig. 14:**
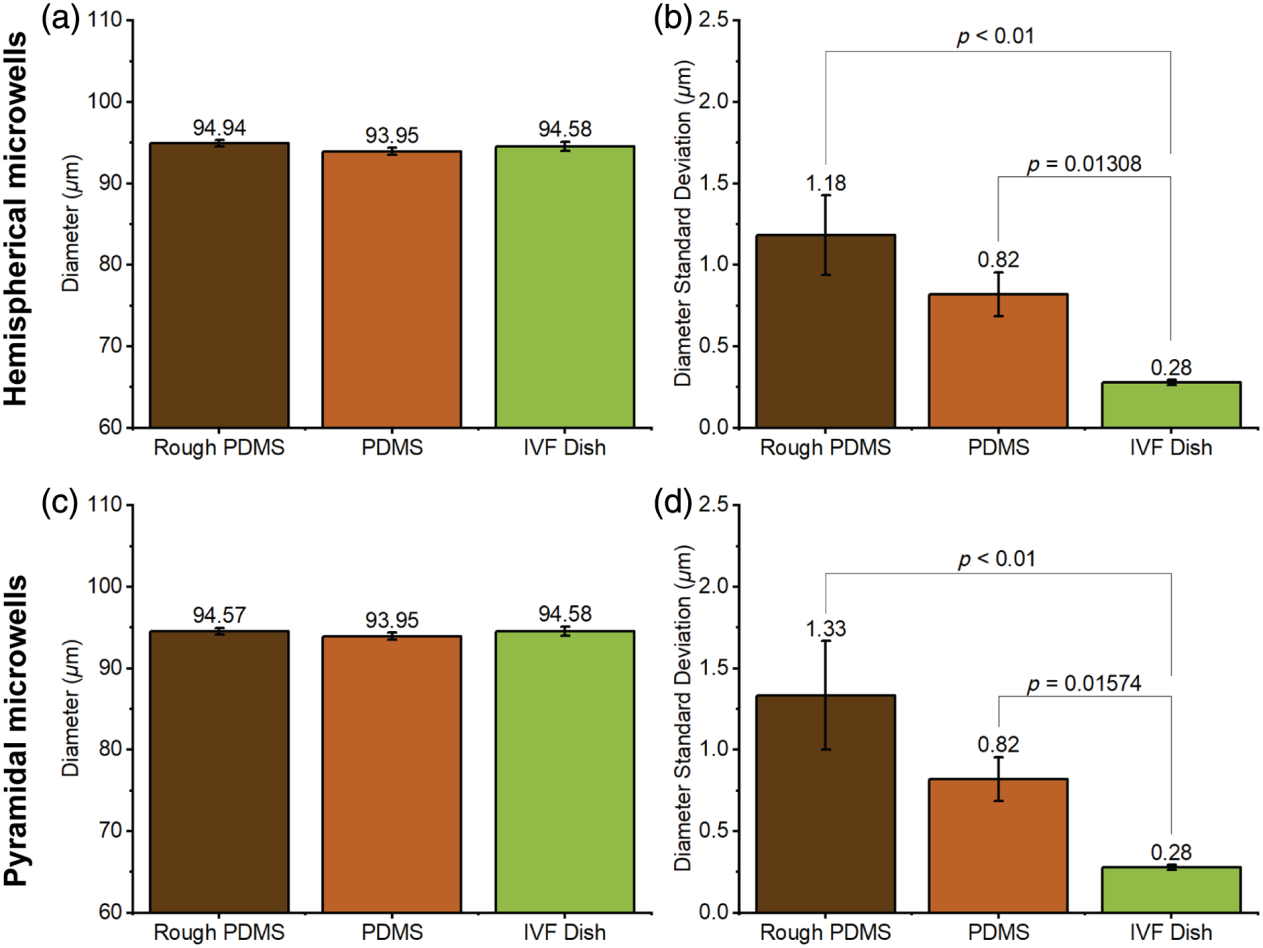
Microsphere diameter and diameter standard deviation measurements in (a), (b) hemispherical and (c), (d) pyramidal microwells (n=9).

To assess the level of distortion introduced by the roughness of the 3D-printed mold, we compared the microsphere diameters and their standard deviations in different culture dishes. The results are summarized in Fig. 14. No statistical significance was found in the diameters for hemispherical and pyramidal microwells when tested by Kruskal–Wallis’s one-way ANOVA, indicating that the roughness does not affect the diameter. Statistical significance was found between rough PDMS and IVF dish as well as PDMS and IVF dish, when tested by Kruskal–Wallis’s one-way ANOVA, indicating rough surface causes poor uniformity in the circular shape of the microspheres. Although no significance was found between rough PDMS and PDMS, the average standard deviation of the diameter of the rough PDMS was higher than PDMS, indicating that the higher surface roughness caused a distorted circular shape of the microspheres.

### 3.6 Optical Aberrations Assessment on WOW Dishes of Different RIs

**Fig. 15:**
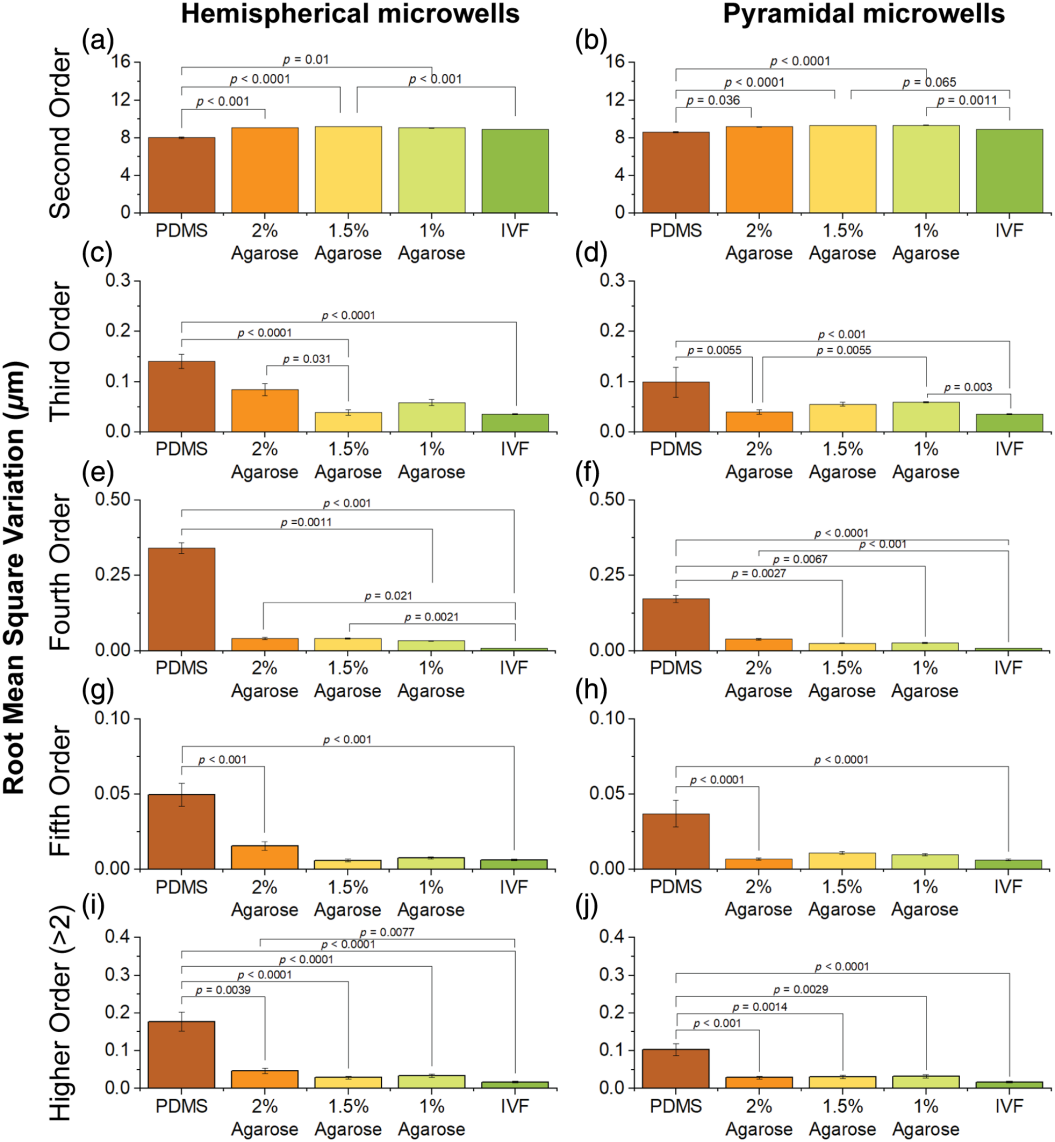
Zernike root mean square (RMS) wavefront error for hemispherical (left column) and pyramidal (right column) microwells fabricated from PDMS, agarose (2%, 1.5%, 1%), and IVF control dishes (n=9): (a)–(b) second-order aberrations (defocus and astigmatism); (c)–(d) third-order aberrations (coma and trefoil), (e)–(f) fourth-order aberrations (primary spherical aberration, secondary astigmatism, and tetrafoil), and (g)–(h) fifth-order aberrations (secondar coma, secondary trefoil, and pentafoil); (i)–(j) aggregated higher-order RMS (sum of Zernike orders >2).

Wavefront analysis is summarized in Fig. 15. Pairwise comparisons with statistically significant differences (Kruskal–Wallis one-way ANOVA) are indicated in the figure.

Second-order Zernike terms—defocus and primary astigmatism [Figs. 15(a) and 15(b)]—exhibited relatively large RMS variations (≈8−10  μm) across all materials, including the IVF dish. Notably, substantial second-order error was present even when no microwell (i.e., in the IVF dish condition) was used. Because brightfield transillumination introduces unavoidable angular illumination—even with a nearly closed condenser aperture—these second-order terms most likely reflect illumination-induced defocus intrinsic to the inverted microscope rather than microwell-dependent aberrations. Although some pairwise comparisons reached statistical significance, the absolute differences were small relative to baseline and did not indicate meaningful, material-dependent changes in defocus. Overall, second-order differences were modest compared with the behavior of higher-order terms.

By contrast, third-order aberrations—including coma and trefoil [Figs. 15(c) and 15(d)]—displayed pronounced material dependence. PDMS microwells generated the highest RMS variation in both hemispherical and pyramidal geometries, significantly greater than the IVF dish. Agarose microwells showed a concentration-dependent reduction in third-order error, with 2% and 1.5% agarose producing RMS variations statistically indistinguishable from the IVF dish.

Fourth-order aberrations—including primary spherical aberration, secondary astigmatism, and tetrafoil [Figs. 15(e) and 15(f)]—further differentiated material performance. PDMS microwells produced substantially larger fourth-order RMS variations than agarose or IVF dish conditions. Significant differences were observed between PDMS and 1% agarose, as well as between PDMS and IVF dishes, for both microwell shapes. Agarose microwells—particularly the 1% and 1.5% formulations—exhibited very low fourth-order aberrations, with 1% agarose statistically indistinguishable from the IVF dish.

Similarly, fifth-order aberrations—including secondary coma, secondary trefoil, and pentafoil [Figs. 15(g) and 15(h)]—were highest in PDMS microwells and minimal in agarose microwells and IVF dishes. Significant differences were observed between PDMS and 2% agarose and between PDMS and the IVF dish for both microwell geometries. Agarose microwells again showed small aberrations, with 1% and 1.5% agarose yielding very low fifth-order RMS variations comparable to the IVF dish.

To capture overall optical performance beyond defocus, we summed the higher-order Zernike terms [>2nd order; Figs. 15(i) and 15(j)]. PDMS microwells produced the largest total higher-order RMS variations—significantly greater than all other conditions—whereas agarose microwells, especially 1% and 1.5% agarose, displayed RMS variations comparable to the IVF dish with no significant difference.

### 3.7 Agarose Stability

**Fig. 16:**
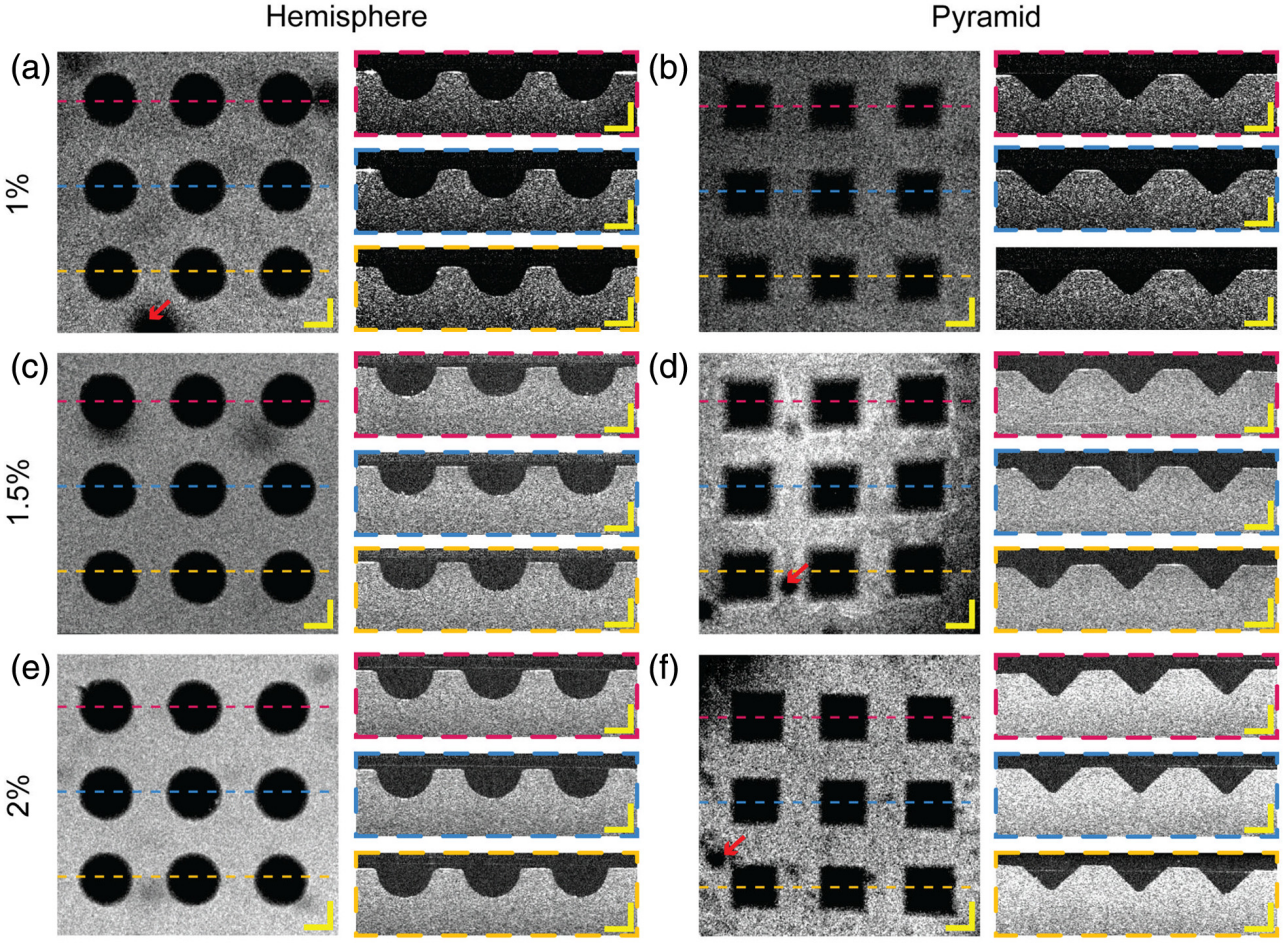
OCT images of the three replications of the agarose microwell array. Images of the hemispherical microwell array and pyramidal microwell array of 1% (a), (b), 1.5% (c), (d), and 2% (e), (f) agarose, including averaged intensity z-projections and cross-sectional images (scale bar: 200  μm). The black spots marked with red arrows are caused by the floating oil bubbles that block the imaging beam.

En face and side view images of fabricated microwells inspected by OCT after six-day culture are shown in Fig. 16, where the left and right columns show the hemispherical and pyramidal microwells, respectively, and rows show the concentration of 1%, 1.5%, and 2% agarose. The black spots indicated by red arrows are caused by floating oil bubbles that block the imaging beam. Table 2 summarizes the observed dimensions. We observed slight deformations in 1.5% and 2% pyramidal microwells, as seen in en face images. All the microwell shapes were retained, as shown in the side-view images. Quantitatively, we observed an increment in the diameter and side length of hemispherical and pyramidal microwells in 1% agarose. In summary, the agarose showed decent stability after 6-day culture.

**Table 2:** Dimension characterization of hemispherical and pyramidal microwells in an agarose microwell array after six-day culture (number of microwells, n=9, SEM, standard error of mean).

	Hemispherical microwell		Pyramidal microwell	
	Diameter (mean ± SEM, mm)	Depth (mean ± SEM, mm)	Side length (mean ± SEM, mm)	Depth (mean ± SEM, mm)
1%	406.5 ± 2.3	198.5 ± 0.9	407.0 ± 2.6	175.3 ± 1.7
1.5%	403.5 ± 2.5	200.7 ± 1.5	382.0 ± 3.8	168.2 ± 2.0
2%	399.2 ± 1.8	200.5 ± 0.7	385.2 ± 1.4	174.9 ± 1.2

### 3.8 Mouse Embryo Imaging

**Fig. 17:**
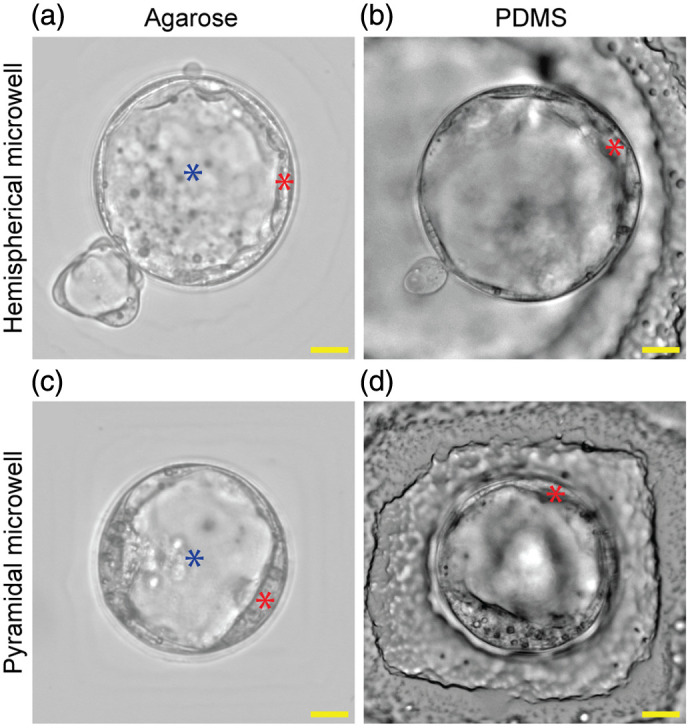
Representative mouse embryo images taken in 1.5% agarose microwells in this work and polydimethylsiloxane (PDMS) microwells in our previous work^18^ by brightfield microscopy: (a), (b) mouse blastocyst in agarose and PDMS hemispherical microwells; (c), (d) mouse blastocyst in agarose and PDMS pyramidal microwells (blastocyst cavity marked by blue star; trophectoderm marked by red star; scale bar: 20  μm).

Figures 17(a) and 17(c) show representative mouse embryos imaged with BF in a hemispherical microwell and a pyramidal microwell, respectively. To compare the imaging quality, mouse embryo images taken in a PDMS microwell developed in our previous work^18^ are placed next to embryo images in agarose microwells in Figs. 16(b) and 16(d). With the reduced appearance of ridges, clearer visualization of the blastocyst cavity [marked by a blue star in Figs. 17(a) and 17(c)] is achieved compared with the PDMS microwells. We also observe the clear trophectoderms (TEs) in agarose microwells [marked by a red star in Figs. 16(a) and 16(c)] that are hard to assess in PDMS microwells. In summary, using the agarose WOW dish, embryos successfully developed to blastocyst on day 7 (embryonic day 4.5), suggesting that the agarose WOW dishes can support embryo culture, and the imaging quality was improved to reveal the morphology of embryos.

## 4 Conclusion

We have demonstrated a method to fabricate index-matching 3D WOW dishes that can improve the imaging quality of embryos viewed directly in microwells. The material used for this demonstration, agarose, was shown to support embryo growth. A comprehensive toxicity study, which is beyond the scope of this paper, should be undertaken to establish readiness for clinical translation.

We confirmed the reproducibility of our fabrication method by comparing the dimensions of three replications. The application of a low-RI material improves the optical resolution compared to high-RI materials for embryo imaging. Geometric distortion of pyramidal and hemispherical microwells as measured by calibrated spherical beads shows improvement in the accuracy of bead diameters for low-RI (i.e., agarose) WOW dishes compared with high-RI (i.e., PDMS) WOW dishes. Clinically, this improvement is important because embryo grading and selection, which are essential steps during IVF, rely on high-quality embryo images. We also verified that the agarose microwells do not significantly deform over six days of incubation, indicating that agarose is a feasible material for embryo culture. In addition, we performed a wavefront-sensor–based aberration assessment to further evaluate the optical performance of the microwell materials. This analysis revealed clear differences in higher-order Zernike terms (i.e., aberrations) across PDMS and agarose WOW dishes. PDMS microwells produced larger third- through fifth-order aberrations, consistent with the geometric distortion observed in calibration bead measurements. By contrast, low-RI agarose microwells generated aberration levels comparable to the IVF dish, indicating that the reduced refractive-index mismatch helps preserve image quality.

In this study, we were not able to directly measure field curvature because an appropriate calibration target could not be accommodated inside the microwell. Instead, the bead diameter measurements served as an indirect indicator of field curvature effects. Future work will require the development of a suitable in-well target to enable direct characterization of field curvature. We also found that the embryos are very sensitive to the fabrication procedures. In our current process, fabricated agarose WOW dishes had to be freshly made before each use, as we have not yet identified a reliable method to keep agarose arrays sterile without dehydration in the long term. Nevertheless, as precast agarose gel is available for DNA electrophoresis, we believe that this method may be used in the future to preserve agarose for making WOW dishes in advance. In addition, the current fabrication process is time-consuming and labor-intensive, which limits its current application in large-scale research. Future efforts to improve the manufacturing process can simplify the fabrication process and reduce costs. Although our study does not show a negative impact of agarose on embryo culture, it is insufficient on its own to confirm its safety due to the limited sample size. A mouse embryo assay that follows the U.S. Food and Drug Administration (FDA) guidelines must be conducted to confirm the safety of agarose before clinical translation. The need for multiple materials also imposes complexity on the fabrication of WOW dishes and introduces the risk of cytotoxicity, which limits its current application in large-scale research and clinical settings. Although a previous study has shown that PDMS is compliant with mouse embryo assays,^40^ indicating its safety for embryo culture, uncured PDMS could release uncross-linked oligomers to the culture media that may interact with cells or media components.^41^ In addition, different culture media have different RIs. Our study only includes one commercial medium, and further testing is necessary to find optimal agarose concentrations that match the RIs of different media, including those for human culture. The primary goal of this work was to propose the general strategy of RI matching for WOW dish fabrication and compare and report its optical performance. It is possible that better materials that are embryotoxicity-free and of low RI may be identified in the future, which can improve clinical adaptability.

Although 3D printers provide an easy way to produce microwells of flexible shapes, ridges produced by the 3D-printed molds frustrate imaging and compound geometric distortion, as measured by bead diameters. This artifact is a result of the limited axial resolution of 3D printers. Our study showed that the ridges in PDMS WOW dishes produced by a 3D-printed mold affect the uniformity of circular microspheres. Molds produced by a low-resolution 3D printer do not change the microsphere diameter but lead to a higher standard deviation in measured diameters, an indication of higher roughness affecting the image measurement. Advanced techniques that offer sub-micron resolution, such as two-photon polymerization, 3D printing, and laser micro machining, are methods to avoid the 3D print ridges; however, such equipment remains high-cost and, therefore, not accessible for quick prototyping. Our index-matching strategy reduces the presence of the ridges and the need for more advanced techniques.

The use of WOW dishes with 3D microwells has already been shown to improve ART outcomes in prior research; however, 3D microwells introduce aberrations and distortions that hinder the imaging quality, making such dishes impractical for embryo imaging and grading. Therefore, only end-point imaging is available for embryo grading. The current work makes it possible, for the first time, to also use such dishes for imaging throughout the embryo grading process, which is necessary to motivate integration of WOW dishes into the clinical workflow.

In addition, the mechanism of the culture quality enhancement in WOW dishes remains unclear. One potential explanation is that WOW dishes enhance the diffusion kinetics of waste materials and the concentration of autocrine factors around the embryo compared to conventional droplet culture, based on numerical simulation;^42^ however, there is a lack of rigorous studies to investigate how WOW dishes promote the growth of embryos. Our strategy of enabling high-quality imaging of embryos in WOW dishes could enable research that tackles the problem by allowing the observation of subcellular activities directly in the dish.

Integration of WOW dishes into the culture, selection, and grading process can ultimately improve ART outcomes.

## Supplementary Material

10.1117/1.BIOS.3.1.012103.s01

## Data Availability

The code and data developed in this study are available from the corresponding author upon reasonable request.
