# Supplementary material for: Index matching improves the imaging quality of 3D well-of-the-well dishes for embryo culture
Source: Biophotonics Discov. 2026 Jan 7;3(1):012103. doi: 10.1117/1.BIOS.3.1.012103 (PMC13052483; doi:10.1117/1.BIOS.3.1.012103)
Supplement: Supplementary file 1 [file BIOS_003_012103_SD001.docx]

Supplemental Material for “Sample m Index matching improves the imaging quality of 3D well-of-the-well (WOW) dishes for embryo culture”

Yunqin Zhao,^a,b^ Mark Mc Veigh,^c^ Leon M. Bellan,^b,d^ Audrey K. Bowden^a,b,e,*^

aVanderbilt University, Vanderbilt Biophotonics Center, Department of Biomedical Engineering, Nashville, TN, United States, 37232

bVanderbilt University, Department of Biomedical Engineering, Nashville, TN, United States, 37232

cVanderbilt University, Interdisciplinary Materials Science Program, Nashville, TN, United States, 37232

dVanderbilt University, Department of Mechanical Engineering, Nashville, TN, United States, 37232

eVanderbilt University, Department of Electrical and Computer Engineering, Nashville, TN, United States, 37232

Figure S1. Drawing of the macrowell insert mold.


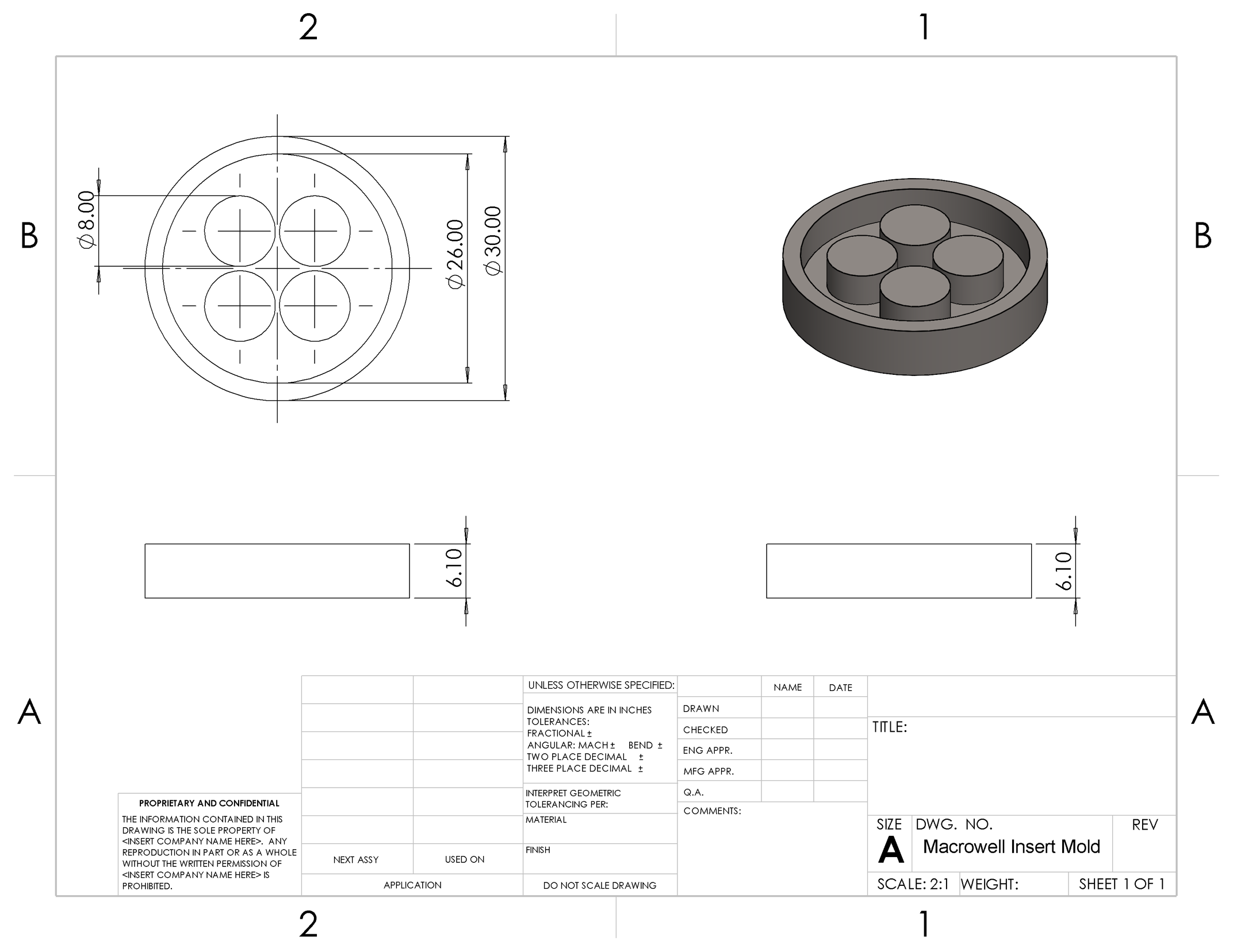


(unit: mm)

Figure S2. Drawing of the microwell array mold.


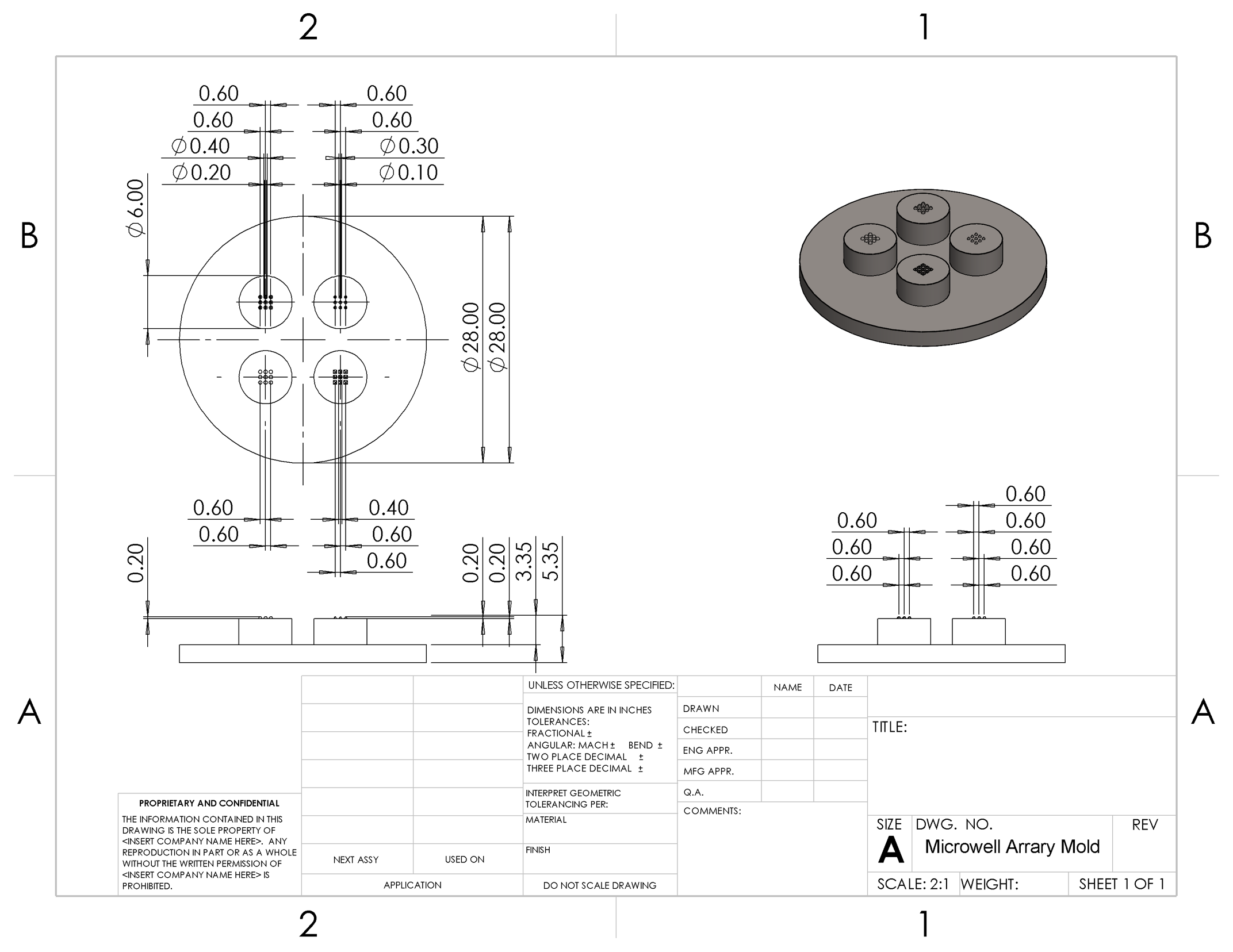


(unit: mm)
